# Proximity proteomics reveals OTUD6B regulation of stress granule dynamics through coalescence with VCP/p97

**DOI:** 10.1038/s41419-026-08451-4

**Published:** 2026-02-06

**Authors:** Dian Yang, Yichao Liu, Yueshun Hong, Enming Miao, Peng Wang, Yuming Sun, Lina Zhou, Shuyan Liu, Yingqiu Zhang, Hongqiang Qin, Mingliang Ye, Han Liu

**Affiliations:** 1 The Institute of Cancer Stem Cell, Dalian Medical University, Dalian, China; 2 State Key Laboratory of Medical Proteomics, Dalian Institute of Chemical Physics, Chinese Academy of Sciences, Dalian, China; 3 State Key Laboratory of Fine Chemicals, School of Chemical Engineering, Dalian University of Technology, Dalian, Liaoning China; 4 Instrumental Analysis Center, Dalian University of Technology, Dalian, China; 5 University of Chinese Academy of Sciences, Beijing, China

**Keywords:** Organelles, Proteomics

## Abstract

Stress granules (SGs) are membrane-less organelles formed through liquid-liquid phase separation of proteins and RNAs, serving as temporary repositories for biomacromolecules to protect cells under stress conditions. Impaired SG disassembly is closely implicated in neurodegenerative diseases and aging, yet the mechanisms regulating SG dynamics are incompletely investigated. The constituents of heterogenous SGs are complicated and broadly categorized as core and shell components. In contrary to the relatively stable core components, our understanding of the diverse SG shell is deficient. By combining interactomic and proximity proteomic approaches, we reveal that the deubiquitinating enzyme OTUD6B is associated with SG-related functions. Immunofluorescence assays showed that OTUD6B localized to SGs, as well as regulated their early assembly and clearance, partially dependent on its enzymatic activity. Further proximity proteomics and interactomics results uncover the ATPase VCP/p97, a key SG disassembly factor, as an OTUD6B-associated protein. OTUD6B and VCP association is governed through their disordered regions normally participated in biomolecular condensation. VCP knockdown or pharmacological inhibition phenocopied OTUD6B silencing by leading to defects in SG dynamics. Mechanistically, SG coalescence of VCP incurred by OTUD6B in a partially enzymatic activity-dependent manner functions to accelerate not only the early assembly, but also SG clearance following stress removal. Therefore, our findings establish OTUD6B as a critical modulator of SG dynamics, linking its function to stress responses and potential disease mechanisms.

## Introduction

Eukaryotic cells have evolved to acquire both membrane-bound and membrane-less organelles (MLOs), allowing them to spatially coordinate diverse biological processes in the highly organized intracellular milieu. MLOs are phase-separated condensates of large biomolecules formed through synergies between network dynamics and density changes [1, 2]. In comparison to membrane-bound organelles, MLOs show enhanced plasticity due to their lack of encapsulating membranes and dynamic morphologies in response to environmental changes. Stress granules (SGs) are condensates that commonly emerge following the inhibition of translation initiation in cells exposed to various stressors, representing a typical stress-responsive MLO [3].

Studies of SG contents uncovered their polysome-derived nature, with the general components of SGs being messenger ribonucleoproteins (mRNPs) released from collapsing polysomes [4]. These mRNPS contain selective mRNA transcripts, translation initiation factors and 40S ribosomal subunits [5, 6]. Key components of SGs also include RNA-binding proteins (RBPs) that are responsible for translational silencing, RNA decay and other RNA metabolism processes [7–9]. Certain proteins without RNA-binding ability have recently been discovered to be integrated into SGs through protein-protein interactions [10, 11]. SGs serve as a temporary repository for intracellular mRNAs to promote cell survival during stress conditions. Upon stress relief, SGs can disassemble through a multi-step process by mechanisms that are not fully understood yet [12]. Impaired disassembly of SGs is an important contributor to various pathological conditions, especially in neurodegenerative diseases, aging and associated diseases [13–15].

Proteomic analyses have revealed that SGs are heterogeneous structures consisting of a relatively stable “core” and a dynamic “shell” component [16]. A subset of RBPs with intrinsically disordered regions (IDRs), such as Ras GTPase-activating protein-binding protein 1 (G3BP1), and translational repressor T-cell intracellular antigen 1 (TIA1), are major SG core components [10]. In contrast to the relatively stable SG cores, frequent matter exchanges with the surrounding milieu contribute to a dynamic composition of the shell. Various shell members play pivotal roles in response to stress signal changes to regulate the dynamics of SGs. For instance, peripheral FAF2 (FAS-associated factor 2) was reported to recruit valosin-containing protein (VCP) and thus trigger the dissolution of SGs through recognizing ubiquitinated G3BP1 [11]. Therefore, discovering novel components will provide insights into the dynamic regulation of SG formation and disassembly, as well as shed light on understanding the pathological features of SG-related diseases.

OTUD6B is a deubiquitinating enzyme (DUB) predicted to contain a conserved catalytic OTU domain and ubiquitin-binding regions at the C-terminus [17, 18]. In mammals, OTUD6B has been shown to be closely associated with cell proliferation, inflammation, and cancer [19–25]. Recent investigations have started to reveal novel biological functions and correlating substrates of OTUD6B. Through its catalytic activity, OTUD6B functions to reduce the ubiquitylation of various substrates, such as β-TrCP, DDX5, KIFC1, IRF3 and LIN28B [20, 25–28]. However, this OTU member can also regulate certain proteins like pVHL independent of DUB activity [24]. Interestingly, recent studies have unraveled the functions of OTUD6B homologs in translation regulation. Otu2, the yeast homolog of OTUD6B, was reported to bind to 40S ribosomes and remove ubiquitin from eS7 to drive translation [29]. This function was also conserved in Drosophila, where OTUD6 deubiquitylates RPS7/eS7 to regulate translation and stresses [30].

Inspired by the intriguing evidence that OTUD6B closely associates with the 40S ribosomes that are major SG components, we hypothesized that OTUD6B possibly participates in SG regulation. Indeed, combined analyses with interactomics and proximity proteomics revealed that OTUD6B-associated proteins were enriched in SGs and RNP regulation. Immunofluorescence assays confirmed SG distribution of OTUD6B, and interference with OTUD6B expression affected both the assembly and disassembly of SGs. Further proteomic analyses discovered that the ATPase VCP/p97 closely associated with OTUD6B. Pharmacological or genetic suppression of VCP/p97 recapitulated the effects of OTUD6B inhibition on SG dynamics. Therefore, our results reveal OTUD6B as a novel SG-related protein, which couples with VCP/p97 to modulate SG dynamics.

## Results

### Proteomic profiling associates OTUD6B with SGs

**Fig. 1:**
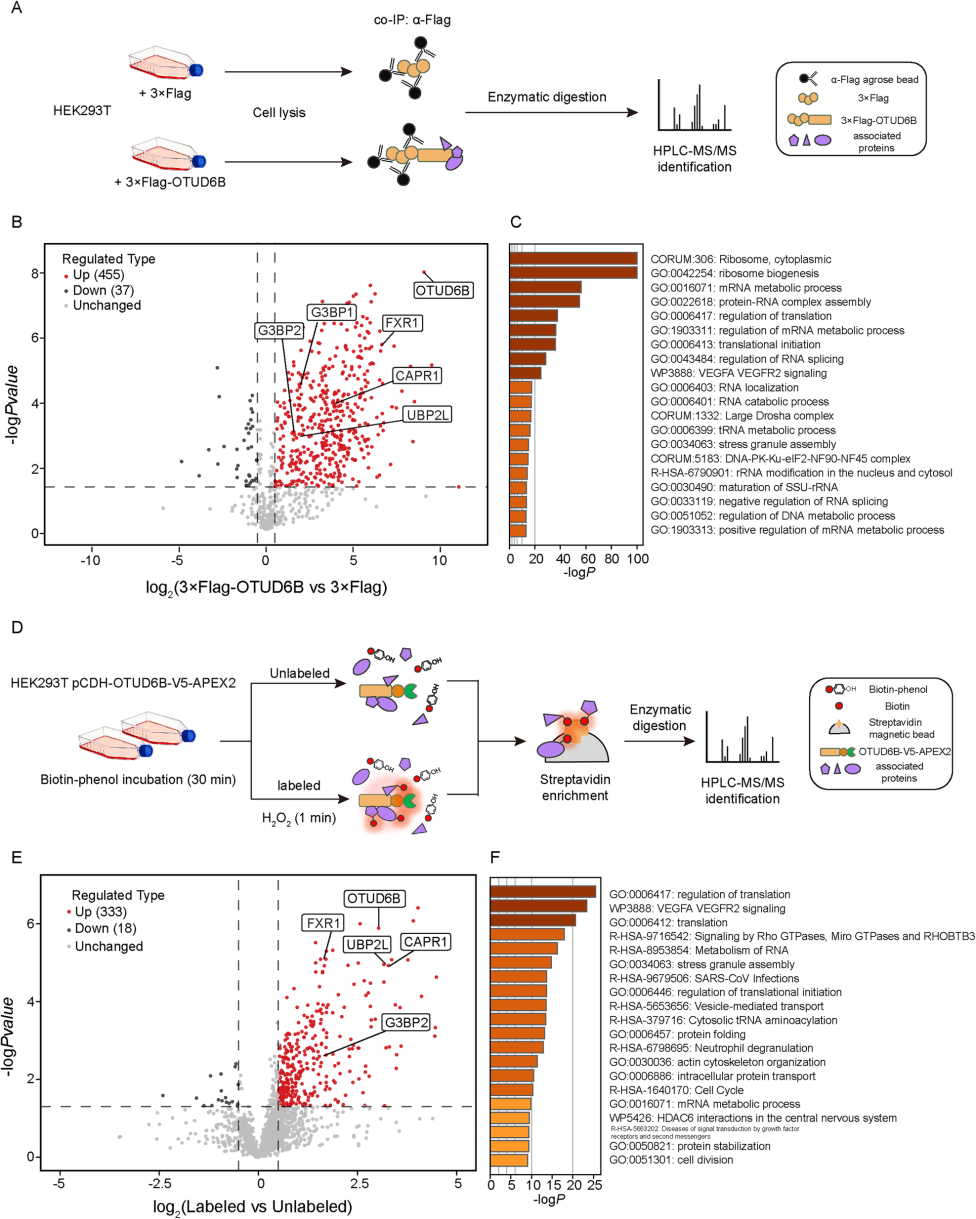
Proteomic strategies uncover the relevance between OTUD6B and SGs. **A** Schematic diagram of co-IP experiments for mass spec-based analyses of OTUD6B-associated proteins. HEK293T cells were transiently transfected with plasmids encoding 3X Flag-tagged OTUD6B or empty vector as control for 48 h. Exogenous OTUD6B was immunoprecipitated with α-Flag antibody from cell lysates. The co-IP samples were digested into peptides for quantitative proteomics analysis. **B** Volcano plot shows significantly enriched candidates in the OTUD6B group (red dots) identified from the mass spec analysis. The thresholds of log_2_|FC|≥ 0.5 and log_10_*P*value ≥ 1.3 are demarcated by dashed lines, *n* = 3 biological independent experiments. OTUD6B and associated core SG proteins are labeled with names. **C** Bar chart of the top-20 enriched pathways of significant OTUD6B-associated proteins analyzed by the Metascape website (https://metascape.org/). Color scheme shows -log transformed *P* values. **D** Schematic diagram of APEX2-based proximity biotinylation labeling experiment for mass spec-based analysis of OTUD6B-neighbouring proteins. For biotinylation, puromycin-selected stable HEK293T-pCDH-OTUD6B-V5-APEX2 cells were pre-incubated in medium containing biotin-phenol (500 μM) for 0.5 h before the labeling reaction. H_2_O_2_ was added to a final concentration of 1 mM to trigger the one-minute labeling reaction. Cells without addition of H_2_O_2_ were used as unlabeled control. Biotinylated proteins were enriched with streptavidin beads from cell lysates. The samples were digested into peptides for quantitative proteomics analysis. **E** Volcano plot shows significant OTUD6B-neighboring proteins (red dots) identified from the mass spec analysis. The thresholds of log_2_|FC|≥ 0.5 and log_10_*P*value ≥ 1.3 were demarcated by dashed lines, *n* = 4 biological independent experiments. OTUD6B and core SG proteins are labeled with names. **F** Bar chart of the top-20 enriched pathways of significant OTUD6B-neighboring proteins revealed by Metascape analysis. Color scheme shows -log transformed *P* values.

We exploited two proteomic approaches to investigate OTUD6B-associated proteins, including co-immunoprecipitation and APEX2-guided proximity labeling. Initially, Flag-tagged OTUD6B transiently expressed in HEK293T cells was immunoprecipitated and processed for mass spec analysis (Fig. 1A). Subsequently, proteins in the vicinity of OTUD6B in HEK293T cells stably expressing APEX2-tagged OTUD6B were labeled by biotinylation through BP and H_2_O_2_ treatment, followed by streptavidin enrichment and mass spec analysis (Fig. 1D). Label-free proteomics was conducted to identify proteins significantly enriched in OTUD6B groups. As shown in the volcano plots (Fig. 1B, E), immunoprecipitation and proximity labeling methods identified 455 and 333 significant interactors, respectively. To delve into the cellular functions of these candidates, we performed enrichment analysis with Metascape [31]. Interestingly, both protein lists were linked to stress granule assembly, in addition to regulation of ribosome and translation as expected (Fig. 1C, F).

**Fig. 2:**
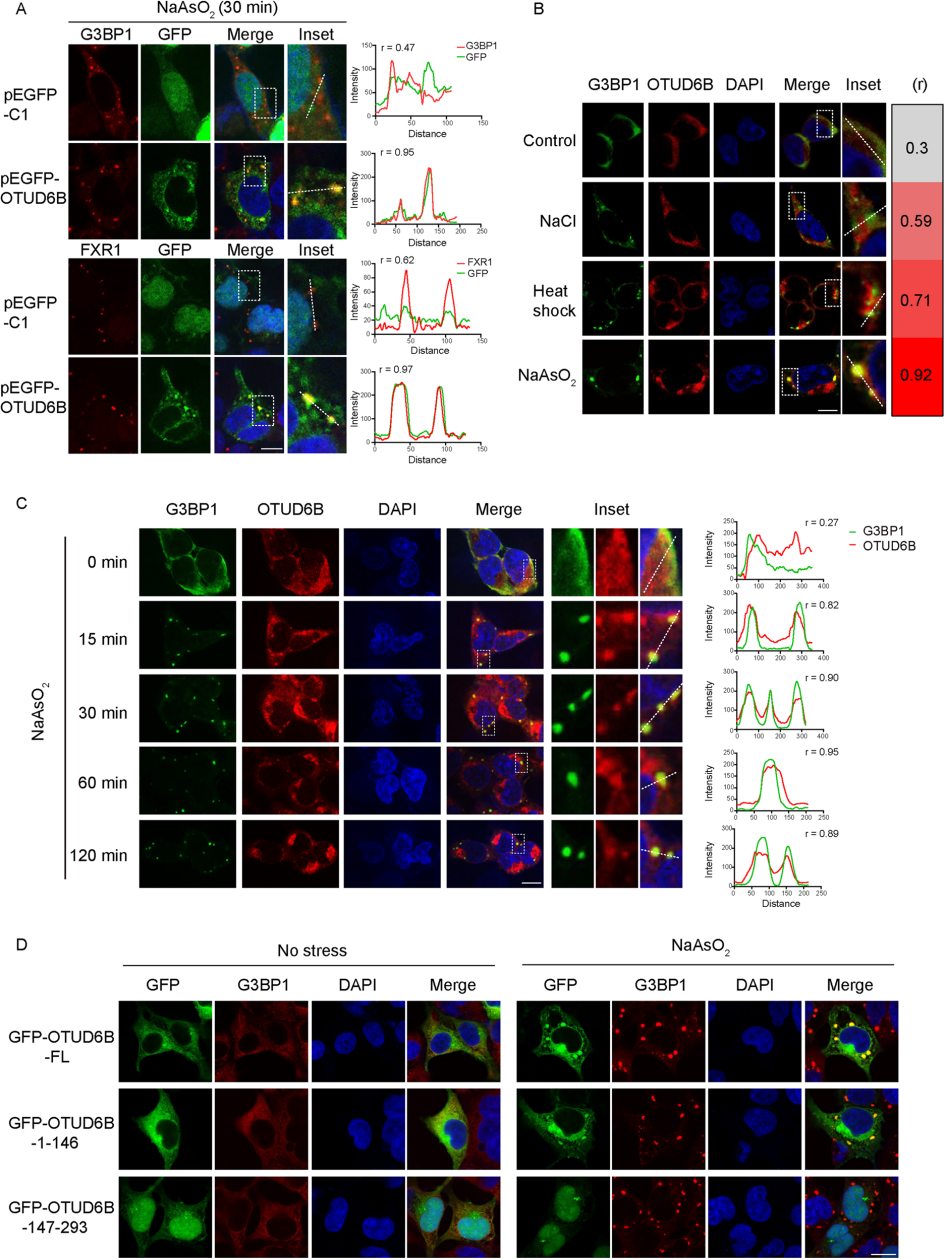
Cellular stresses induce the SG localization of OTUD6B. **A** Representative confocal images show localizations of GFP-tagged OTUD6B or GFP in HEK293T cells from indicated groups co-stained with SG markers (G3BP1 and FXR1). SGs were induced by arsenite treatment (500 μM) for 30 min. Corresponding co-localization analysis of the indicated fluorescence signals with Pearson’s correlation coefficients (r) is presented in the graph on the right. **B** Representative confocal images show the localization of endogenous OTUD6B in HEK293T cells under normal conditions or stimuli of different stressors as indicated. Cells were exposed to osmotic stress (200 mM NaCl), heat shock (43 °C), or oxidative stress (500 μM arsenite) for 0.5 h, 1 h, or 0.5 h, respectively. Cells were fixed for IF staining of OTUD6B (red) and G3BP1 (green), along with DAPI for nucleus staining (blue). Pearson’s correlation coefficient (r) calculated with the indicated dotted line in each inset is shown on the right. **C** Representative confocal images show the co-localization analysis of endogenous OTUD6B with G3BP1 in HEK293T cells that underwent different time periods of arsenite treatment. Cells were stained for OTUD6B (red) and G3BP1 (green). DAPI shows the nucleus (blue). Pearson’s correlation coefficient (r) at each time point was analyzed and presented on the right. Insets in each panel show magnification of the dashed rectangle-circled ROI (region of interest) in each group. The white dotted line in each inset provides signal profiling of fluorescence intensities. **D** Representative confocal images show localizations of ectopically expressed GFP-tagged OTUD6B or OTUD6B truncated mutants in HEK293T cells from the indicated groups. SGs were induced by arsenite treatment (500 μM) for 1 h. Cells were stained for G3BP1 (red). DAPI shows the nucleus (blue). Scale bar, 10 μm.

Given that OTUD6B has not been linked to SGs and both methods revealed such an association, we decided to investigate the potential roles of OTUD6B in SG regulation after validating the co-IP with core SG components G3BP1/2, FXR1, and CAPRIN1 (Supplementary Figs. 1A–C and 5). We then expressed GFP-tagged OTUD6B in HEK293T cells and induced SG formation using arsenite. In contrast to GFP, which localized diffusely, GFP-tagged OTUD6B demonstrated obvious colocalizations with two SG markers, G3BP1 and FXR1 (Fig. 2A). This observation encouraged us to investigate the SG association of endogenous OTUD6B in cells subjected to various stressors. Interestingly, quantification results suggest that all stressors caused increased colocalization of OTUD6B with G3BP1 in a descending order of arsenite, heat shock, and NaCl (Fig. 2B). Therefore, we decided to focus on arsenite- and heat shock-induced SGs in the following investigation. Next, we examined the distribution of endogenous OTUD6B over a 120 min duration following arsenite treatment. Relative to the diffuse staining of OTUD6B and G3BP1 in untreated HEK293T and HeLa cells, both proteins started to form punctate structures that colocalized after 15 min, which lasted through 120 min (Fig. 2C and Supplementary Fig. 1D). In subsequent analysis, we compared the contribution of the N- and C-termini (amino acids 1-146 and 147-293 of OTUD6B, respectively) to its SG distribution, and observed that the N-terminus governed SG localization of OTUD6B following arsenite treatment (Fig. 2D). These results confirm that OTUD6B associates with SGs from the early stage of assembly.

### OTUD6B modulates the dynamics of SG formation

**Fig. 3:**
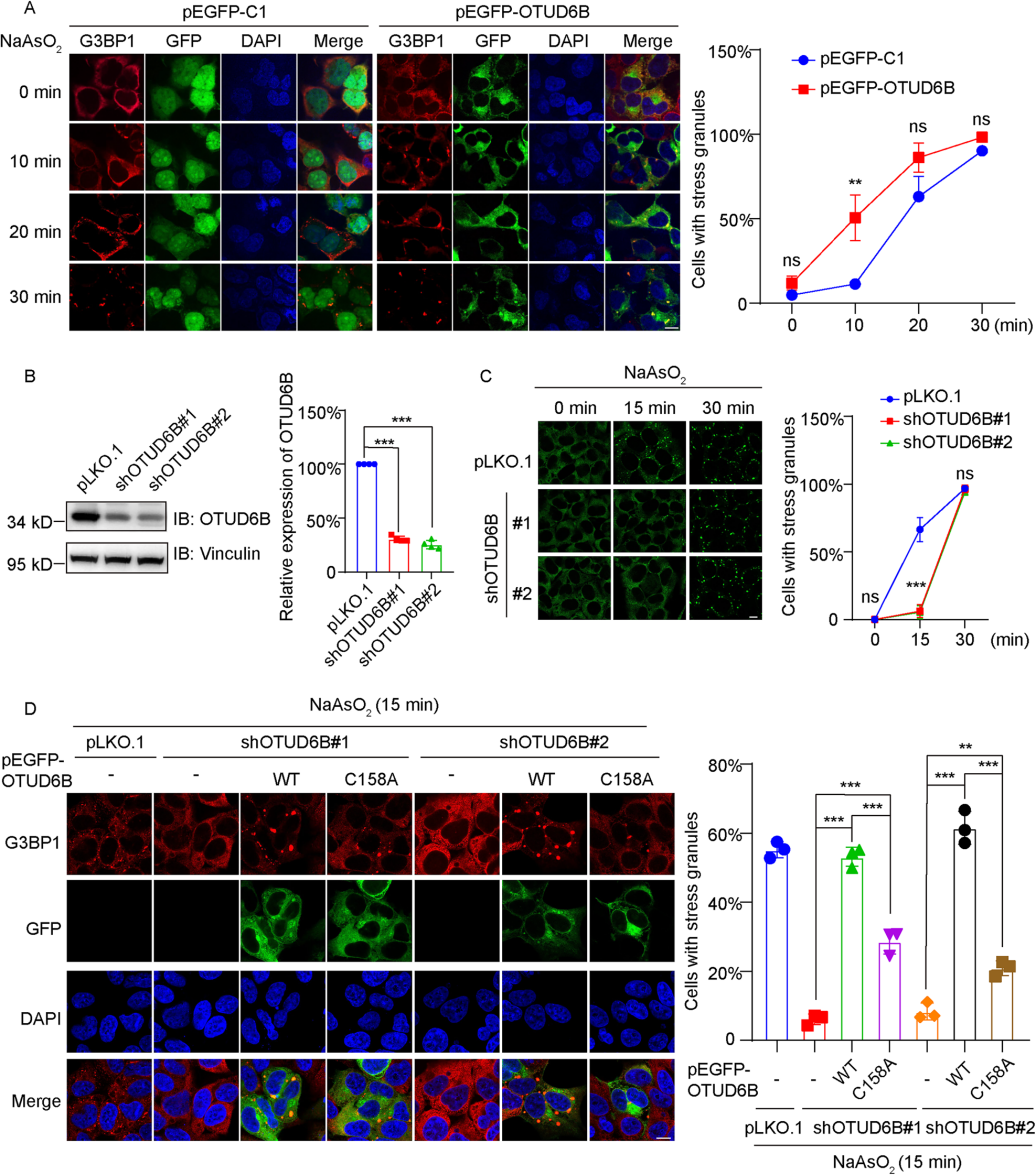
OTUD6B accelerates the early assembly of arsenite-induced SGs. **A** Representative confocal images show the stimulating effects of GFP-tagged OTUD6B on arsenite-induced SG formation in HEK293T cells. HEK293T cells transfected with pEGFP-OTUD6B or empty vector (pEGFP-C1) as control were exposed to the indicated times of arsenite stress (500 μM) before fixation. IF staining of G3BP1 (red) and GFP signals (green), along with DAPI for nucleus (blue) were observed. Quantitation of cells with SGs in GFP-positive cells for each group at indicated time periods was presented in the curves on the right. **B** Representative immunoblot of validation of shRNA-mediated OTUD6B knockdown. Lysates from HEK293T stable cells were analyzed with the indicated antibodies, and quantitation of the relative expression levels of OTUD6B from independent experiments is presented (right, *n* = 4). Vinculin blot was used as a loading control. **C** Representative confocal images show SG formation in stable OTUD6B knockdown cells exposed to arsenite for the indicated times. Fixed cells were stained by G3BP1 (green). Quantitation of cells with SGs for each group from independent experiments (*n* = 3) is shown on the right. **D** Representative confocal images from rescue experiments in stable HEK293T cells. OTUD6B knockdown cells were transfected with either wild-type (WT) or catalytically-dead (C158A) form of GFP-tagged OTUD6B as indicated. SGs were induced by 15-minute exposure of arsenite (500 μM). IF staining of G3BP1 (red) and GFP signals (green), along with DAPI for nucleus staining (blue) were observed. Quantitation of cells with SGs for each group from independent experiments (*n* = 3) is shown on the right. Scale bar, 10 μm. Five random fields of view from each group were used for statistical analysis. Data are shown as mean ± SD. Student’s *t* test was used to calculate *P* values. **P < 0.01, ***P < 0.001, and ns for statistically not significant.

After confirmation of the SG recruitment of OTUD6B, we investigated how changes in OTUD6B expression would affect SG dynamics. We transiently expressed GFP or GFP-tagged OTUD6B and induced SGs with arsenite. By quantifying cells containing G3BP1-positive SGs, we found that OTUD6B overexpression significantly increased SG-containing cells at 10 min, but no significant difference was recorded at later timepoints (Fig. 3A). Using HEK293T and HeLa with stable OTUD6B knockdown, we observed OTUD6B depletion significantly reduced cell numbers with SGs at 15 min but caused no effects at 30 min (Figs. 3B, C and Supplementary Fig. 2A, B and 5). These findings are consistent with results from OTUD6B overexpression, suggesting that OTUD6B promotes the early formation of SGs. To investigate the implication of OTUD6B enzymatic activity in SG regulation, we performed rescue experiments by complementing wild-type or enzymatically inactive OTUD6B (C158A) expression in knockdown cells. As shown in Fig. 3D, wild-type expression recovered SG formation to normal levels, but the C158A mutant only partially increased cell numbers containing SGs. Therefore, these results suggest that OTUD6B enzymatic activity is involved in SG regulation.

Besides arsenite treatment, heat shock is another frequently used method to induce SG formation. Therefore, we investigated the influence of OTUD6B expression on the dynamics of heat shock-induced SGs. Much akin to results obtained with arsenite treatment, overexpression of GFP-tagged OTUD6B also led to significantly increased numbers of cells bearing SGs at 10 min (Supplementary Fig. 3A, B). Consistently, the numbers of SG-containing cells were dramatically reduced after 15 min of heat shock in HEK293T and HeLa cells depleted of OTUD6B (Supplementary Fig. 3C, D). Similarly, in rescue assays with heat shock, the expression of shRNA-resistant OTUD6B wild-type effectively restored SG formation, while the enzymatically inactive C158A only demonstrated partial effects during heat shock stress (Supplementary Fig. 3E). Therefore, these results suggest a common mechanism in the regulation of SG dynamics by OTUD6B under circumstances of oxidative and heat stresses.

### OTUD6B regulates the disassembly of SGs

**Fig. 4:**
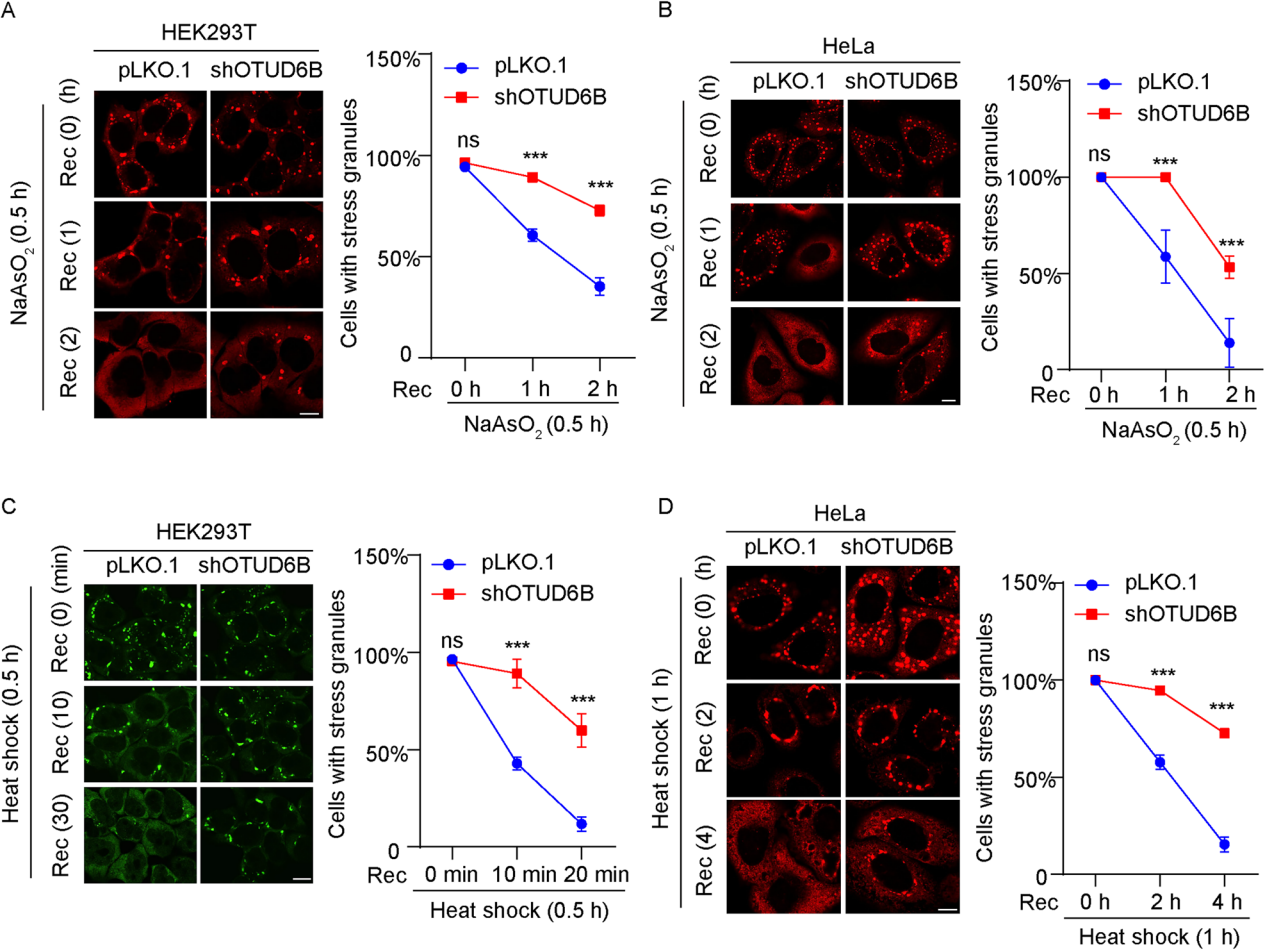
OTUD6B depletion deters SG disassembly following stress removal. **A** Representative confocal images show the IF staining of SG marker G3BP1 (red) in HEK293T cells during the recovery (Rec) from arsenite stress. HEK293T cells stably transfected with shOTUD6B or control pLKO.1 vectors were treated with 500 μM of arsenite for 0.5 h, and then washed to recover in normal medium for 1 and 2 h. Quantitation of the percentages of cells with SGs at the indicated times is shown on the right (*n* = 3). **B** Representative confocal images of G3BP1 staining in stable HeLa cells during the recovery after arsenite stress. Cells with or without OTUD6B depletion (shOTUD6B) were treated as in A. Quantitation of cells with SGs at the indicated times is shown on the right (*n* = 3). **C** Representative confocal images of G3BP1 staining (green) in stable HEK293T cells during the recovery from heat sock. HEK293T cells from A were incubated at 43 °C for 30 min and then returned to 37 °C for 10 and 20 min. Quantitation of cells with SGs at the indicated times is shown on the right (*n* = 3). **D** Representative confocal images of G3BP1 staining (red) in stable HeLa cells during the recovery from heat shock. Stable HeLa cells from B were subjected to a 1-h heat sock at 43 °C before recovery at 37 °C for 2 and 4 h. Quantitation of cells with SGs at the indicated times is shown on the right (*n* = 3). Scale bar, 10 μm. Five random fields of view from each group were used for statistical analysis. Data are shown as mean ± SD. Student’s *t* test was used to calculate *P* values. ***P < 0.001, and ns for statistically not significant.

SGs are highly dynamic structures that emerge in response to cellular stresses, and are normally processed for clearance after stressor removal. Therefore, we investigated the influence of OTUD6B knockdown on the kinetics of SG disassembly. As shown in Fig. 4A, B, following arsenite washout in HEK293T and HeLa cells, G3BP1-positive SGs were readily dissolved in 2 h. However, the depletion of OTUD6B by shRNA significantly delayed these processes, as evidenced by the obvious observation of remaining G3BP1-positive foci after 2 h of recovery. Furthermore, we investigated the effects of OTUD6B knockdown on SG disassembly following recovery from heat shock. In accordance with our findings from arsenite recovery experiments, the depletion of OTUD6B also significantly attenuated the clearance of SGs after stress removal (Fig. 4C, D). Therefore, our results suggest that OTUD6B functions to regulate SG dynamics by affecting both the early formation and the disassembly stages.

### OTUD6B associates with the ATPase VCP/p97

**Fig. 5:**
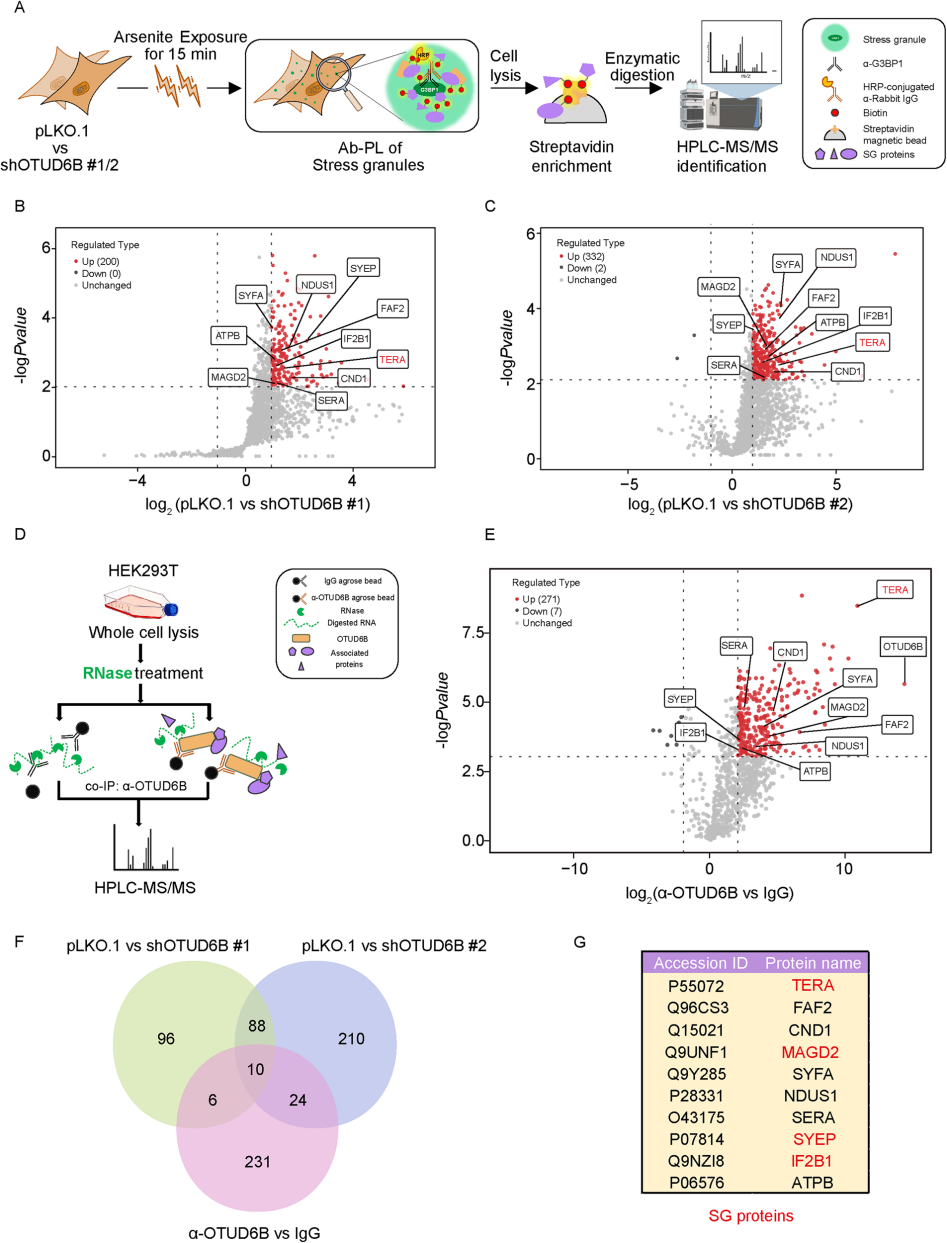
Combined proteomic analyses link VCP/p97 to OTUD6B regulation of SGs. **A** Schematic diagram of an antibody-guided proximity labeling (Ab-PL) experiment for mass spec-based differential SG proteins between OTUD6B knockdown and control cells. SGs were induced by arsenite at 500 μM for 15 min. Fixed cells were treated sequentially with primary α-G3BP1 antibody, HRP-conjugated secondary antibody, and biotin-phenol for labeling reaction. Biotinylated proteins were enriched with streptavidin beads and processed for label-free proteomics. Volcano plots show the differential G3BP1-proximal proteome of control versus shOTUD6B#1 (**B**) or shOTUD6B#2 (**C**). The thresholds of log_2_|FC|≥1 and log_10_*P*value ≥ 2 are demarcated by dashed lines, *n* = 4 independent experiments. SG proteins are labeled with names. **D** Schematic diagram of α-OTUD6B co-IP experiments with RNase treatment for mass spec-based determination of RNA-independent OTUD6B-associated proteins. IgG was used as isotype control. **E** Volcano plot shows the RNA-independent interactome of endogenous OTUD6B (red dots) identified by the RNase-treated α-OTUD6B co-IP experiments in (**D**). The thresholds of log_2_|FC|≥2 and log_10_*P*value ≥ 3 are demarcated by dashed lines, *n* = 4. **F** Venn diagram shows the overlaps from 3 differential proteomic datasets. **G** The list shows 10 overlapped candidates, with SG proteins designated in red.

Inspired by the versatile functionality of OTUD6B in regulating SG dynamics, we next sought to delve into the underlying mechanism of OTUD6B regulation of SGs. To this end, we performed G3BP1 antibody-guided proximity labeling of SG proteins followed by streptavidin enrichment and mass spectrometry (Fig. 5A). Through quantitatively surveying the proteins associated with G3BP1 in control and OTUD6B-depleted cells, we aimed to disclose the differences in recruitment to SGs following 15 min of arsenite treatment, when we observed marked differences with OTUD6B knockdown. As demonstrated in Fig. 5B, C OTUD6B knockdown by both shRNAs dramatically reduced the numbers of proteins enriched following G3BP1 antibody-guided proximity biotinylation, confirming the disruptive effects of OTUD6B knockdown on SG protein recruiting. Considering that various RNA species play pivotal roles in SG assembly [32–35], and our proteomic analysis indeed identified a number of RNA-associated proteins, we repeated mass spectrometric analysis of OTUD6B immunoprecipitation with the addition of RNase to remove RNA-mediated interactions to focus on protein binders. Therefore, using an antibody against OTUD6B and control IgG, we conducted immunoprecipitation assays followed by label-free proteomics and thus obtained a list of potential candidates that associated with OTUD6B in an RNA-independent manner (Fig. 5D, E).

In order to pinpoint key OTUD6B interactors, we performed Venn diagram analysis to uncover overlapped candidates from proximity proteomics and OTUD6B interactome data. As shown in Fig. 5F, G 10 candidates appeared in the overlaps from 3 datasets, which include 4 SG-related proteins (TERA, MAGD2, SYEP and IF2B1). Transitional endoplasmic reticulum ATPase (TERA), commonly known as VCP or p97, plays important roles in the regulation of organelle functions, including the Golgi apparatus, endoplasmic reticulum and SGs. VCP association with G3BP1 was markedly suppressed by OTUD6B knockdown as revealed by proximity labeling analysis (Fig. 5B, C), and it was identified as the second most enriched protein after OTUD6B in the immunoprecipitation analysis (Fig. 5E). Therefore, we decided to focus on VCP to investigate its implication in OTUD6B regulation of SGs.

### OTUD6B regulates SGs through VCP

**Fig. 6:**
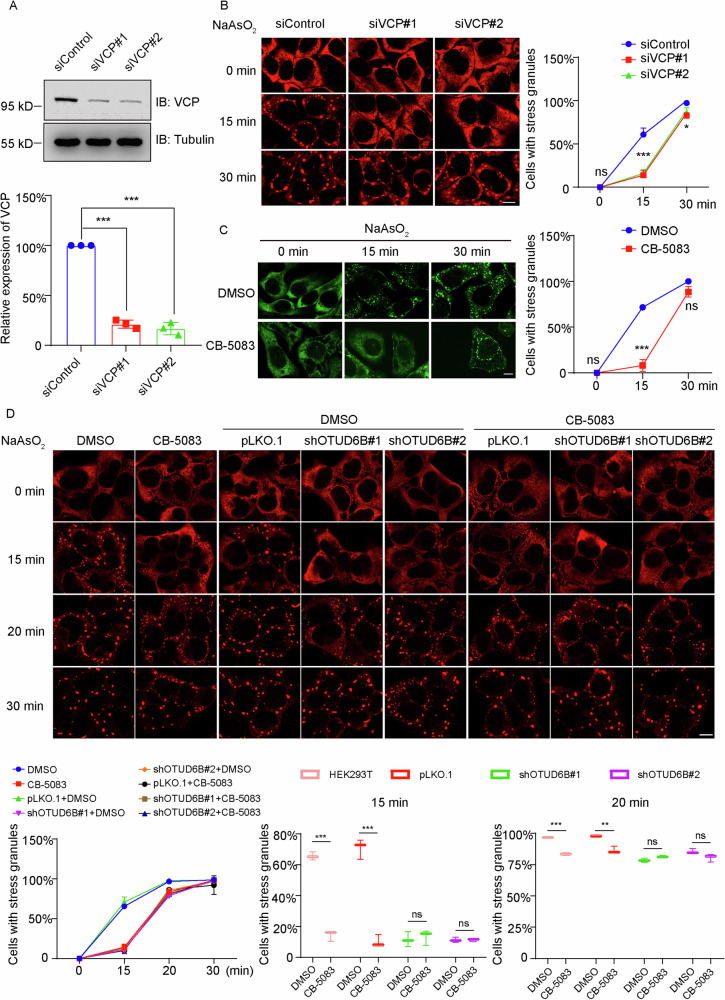
VCP suppression phenocopies OTUD6B knockdown, leading to deterred SG assembly. **A** Representative immunoblot shows shRNA-mediated OTUD6B silencing in HEK293T cells. Column chart below shows quantitation of the relative expression levels of VCP from independent experiments (*n* = 3). Tubulin blot serves as a loading control. **B** Representative confocal images demonstrate SG formation by G3BP1 staining in HEK293T cells with or without VCP silencing with two siRNAs as indicated. Cells were exposed to the indicated times of arsenite (500 μM). Quantitation of cells with SGs for each group from independent experiments (*n* = 3) is shown on the right. **C** Representative confocal images show SG formation in HeLa cells with pharmacological inhibition of VCP. Cells were pretreated with DMSO or CB-5083 (5 μM) for 4 h, before exposed to 15 or 30 min of arsenite stress (500 μM). Quantitation of cells with SGs at the indicated time periods for each group from independent experiments (*n* = 3) is presented on the right. **D** Representative confocal images show SG formation in HEK293T cells with or without stable OTUD6B knockdown in the presence of CB-5083 or DMSO as vehicle control. Cells were pretreated with DMSO or CB-5083 (5 μM, 4 h), and then subjected to arsenite stress (500 μM) for the indicated time periods. Cells were fixed for IF staining of G3BP1. Quantitation of cells with SGs at the indicated time periods for each group from independent experiments (*n* = 3) is shown below. Scale bar, 10 μm. Five random fields of view from each group were used for statistical analysis. Data are shown as mean ± SD. Student’s t-test was used to calculate *P* values. *P < 0.5, ***P < 0.001, ns for statistically not significant.

VCP has been shown to regulate the disassembly of SGs through engaging with ubiquitylated SG proteins [11]. To investigate the effects of VCP on the early stage of SG formation, we knocked down VCP in HEK293T cells using two siRNAs, which effectively depleted VCP protein levels (Figs. 6A and Supplementary Fig. 5). We then induced SG formation in these knockdown and control cells using arsenite and heat shock. As shown in Figs. 6B and 4A, siRNA-mediated VCP silencing led to markedly reduced SG formation at 15 min of stress exposure, although the differences were gradually decreased by 30 min. Furthermore, we confirmed the activity of VCP in regulating SG formation at early stages using its ATPase activity inhibitor CB-5083. As shown in Fig. 6C and Supplementary Fig. 4B, SG formation was induced with arsenite or heat shock in HeLa cells in the presence or absence of CB-5083. In accordance with observations using siRNA-mediated knockdown, CB-5083 treatment resulted in markedly reduced numbers of SGs after 15 min of stress treatment. Therefore, these results collectively suggest that VCP inhibition demonstrated similar effects on SG formation with OTUD6B knockdown by showing strong suppression at 15 min of stress exposure.

Next, we set out to test whether there are synergistic or additive effects of OTUD6B and VCP inhibition on SG formation. Therefore, we treated HEK293T cells stably transfected with control vector or OTUD6B shRNAs using arsenite or heat shock in the absence or presence of CB-5083. Quantification results showed that OTUD6B knockdown did not further enhance the inhibitory effects of CB-5083, with combined treatment demonstrating similar suppression with single treatment of either OTUD6B knockdown or VCP inhibition (Fig. 6D and Supplementary Fig. 4C). Therefore, our findings suggest that OTUD6B likely regulates SG formation through recruiting VCP.

### OTUD6B recruits VCP to SGs

**Fig. 7:**
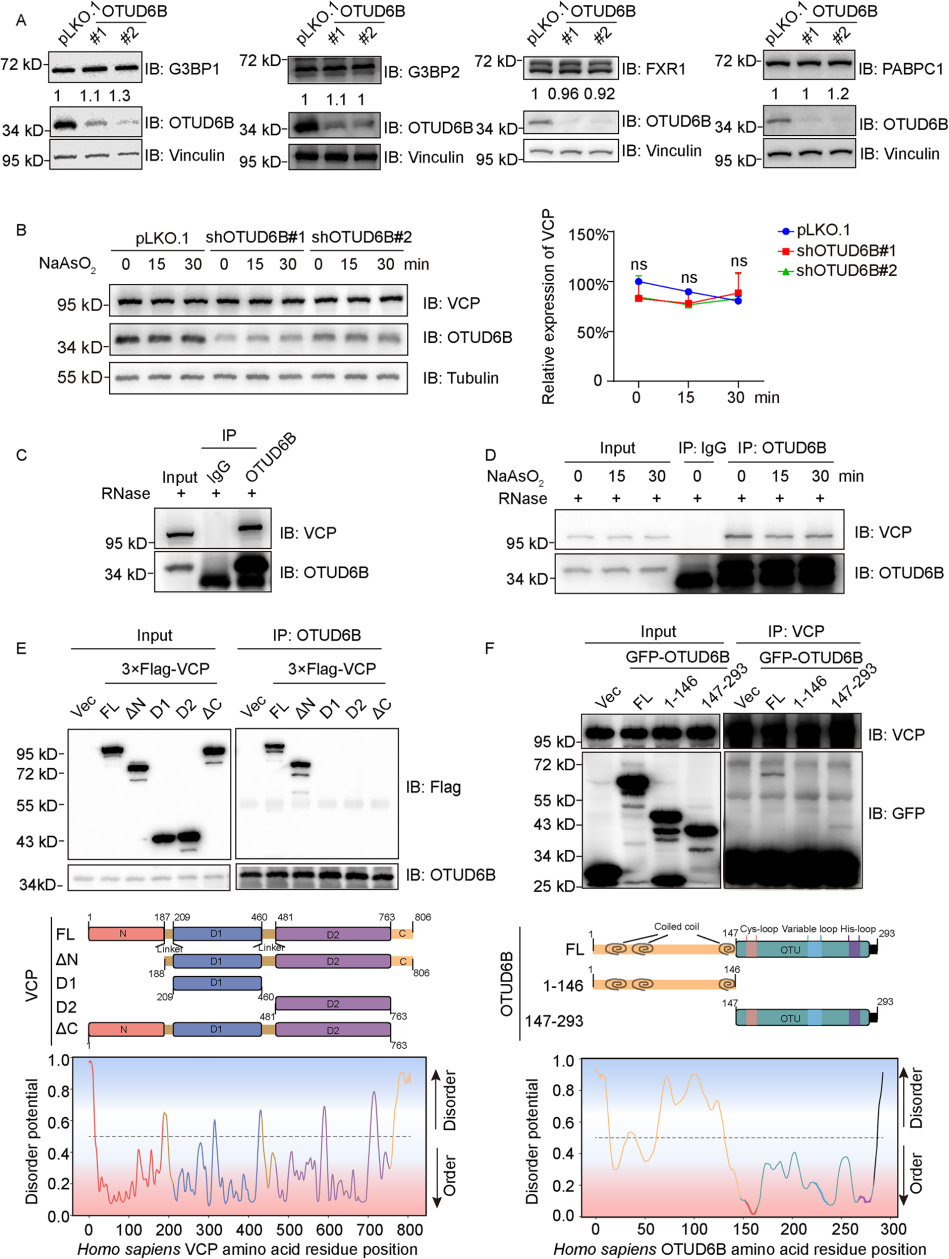
OTUD6B associates with VCP through disordered regions. **A** Immunoblots with lysates from HEK293T cells stably transfected with OTUD6B shRNA (#1 and #2) or vector control pLKO.1 constructs. The relative expression levels of indicated SG proteins (G3BP1, G3BP2, FXR1, and PABPC1) were quantified and labeled beneath corresponding bands. OTUD6B blots show knockdown efficiency, and Vinculin was probed to confirm equal loading. **B** Representative immunoblots with lysates from indicated HEK293T cells either untreated or treated with arsenite for 15 and 30 min. Quantitation of VCP expression levels relative to Tubulin for each group from independent experiments is shown on the right. **C** Immunoprecipitation (IP) assays were performed with α-OTUD6B and IgG isotype antibodies using HEK293T lysates in the presence of RNase. Samples were subjected to immunoblotting assay with α-VCP and α-OTUD6B antibodies. **D** Immunoprecipitation assays using α-OTUD6B and IgG isotype antibodies with lysates from HEK293T cells treated with arsenite for indicated times in the presence of RNase. Samples were subjected to immunoblotting assay with α-VCP and α-OTUD6B antibodies. **E**, **F** Schematic diagrams demonstrate the domain structures and truncated mutants of VCP and OTUD6B as indicated. Below graphs show corresponding analyses of disorder potentials using the web-based PrDOS (https://prdos.hgc.jp/cgi-bin/top.cgi). Co-immunoprecipitation assays using 3X Flag-tagged VCP constructs and GFP-tagged OTUD6B truncations as indicated. HEK293T cells were transiently transfected with empty vector (Vec), full length (FL), or truncation constructs. Cell lysates were incubated with α-OTUD6B or α-VCP antibodies. Immunoprecipitation samples were analyzed by immunoblotting with the indicated antibodies. Data are shown as mean ± SD. Student’s *t* test is used to calculate *P* values; ns stands for statistically not significant.

As DUB frequently influences protein abundance through regulating their stability, we examined the protein levels of several key SG proteins in OTUD6B-depleted cells. As shown in Fig. 7A and Supplementary Fig. 5 SG components G3BP1, G3BP2, FXR1, or PABPC1 did not respond to changes in OTUD6B expression. Similarly, OTUD6B knockdown showed no effects on steady state levels of VCP, nor its abundance with stress treatment (Fig. 7B and Supplementary Fig. 5). However, immunoprecipitated OTUD6B effectively pulled down endogenous VCP under steady state conditions in HEK293T cells (Fig. 7C and Supplementary Fig. 5). Furthermore, with arsenite treatment, VCP also efficiently co-IP with endogenous OTUD6B in the presence of RNase (Fig. 7D and Supplementary Fig. 5). Therefore, these observations corroborated that OTUD6B is constitutively associated with VCP in an RNA-independent manner.

To further investigate the interaction of VCP with OTUD6B, we generated a range of truncation mutants of VCP and OTUD6B (Fig. 7E, F). Results from co-IP assays showed that the very C-terminus of VCP (aa 763-806) that is predicted to be disordered is required for efficient association with OTUD6B [36], while both N- and C-termini of OTUD6B are predicted to contain disordered sequences and are involved in VCP association (Fig. 7E, F and Supplementary Fig. 5). Therefore, these findings suggest that the interaction between OTUD6B and VCP is likely mediated through their disordered regions.

**Fig. 8:**
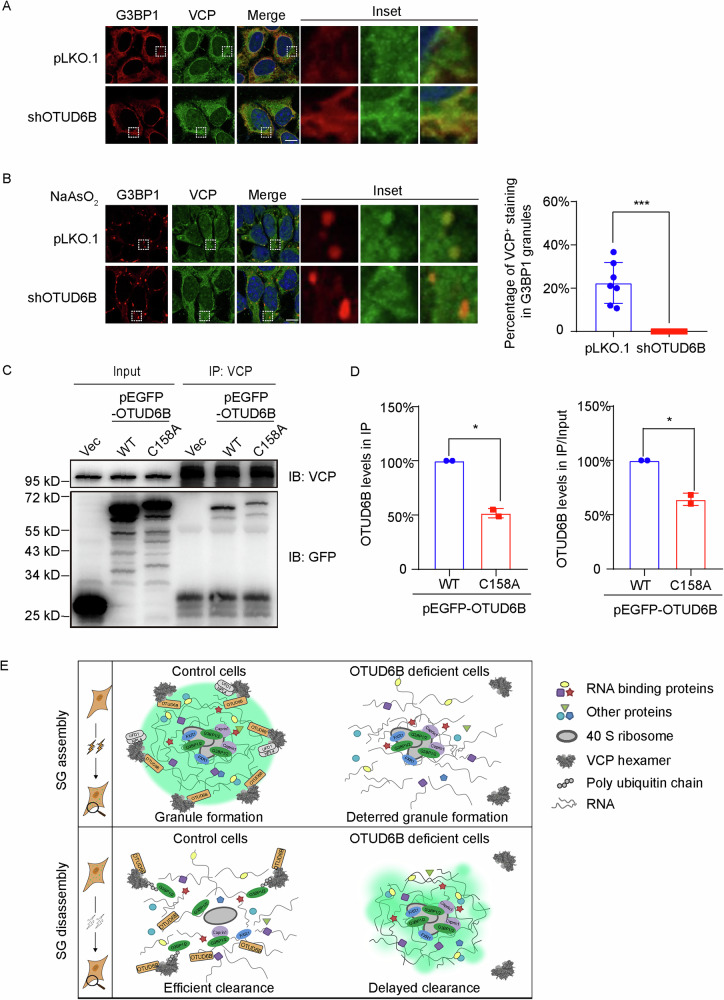
OTUD6B regulates SG dynamics through recruitment of VCP. **A** Representative confocal images show the distribution of G3BP1 and VCP in HEK293T cells stably transfected with pLKO.1 or shOTUD6B constructs. **B** Representative confocal images show the staining of G3BP1 and VCP in HEK293T cells stably transfected with pLKO.1 or shOTUD6B constructs under arsenite stress (500 μM, 45 min). Quantitation of VCP^+^ staining in SGs identified by G3BP1 punctate structures for each group is shown on the right. Seven random fields of view from each group were used for statistical analysis. Inset in each panel shows magnified views of dashed rectangle-circled ROI (region of interest). Scale bar, 10 μm. **C** HEK293T cells were transfected with empty vector (Vec), GFP-tagged wild-type OTUD6B (WT) or catalytically inactive C158A constructs. Immunoprecipitation (IP) was conducted using α-VCP antibody and samples were analyzed by immunoblotting with indicated antibodies. **D** Quantitation of WT and C158A versions of OTUD6B co-immunoprecipitated with VCP in each group, including levels in IP samples and relative levels after ratioing to input. Data are shown as mean ± SD. Student’s *t* test was used to calculate *P* values. *P < 0.05 and ***P < 0.001. **E** A working model demonstrates the regulation of SG assembly and disassembly by OTUD6B through facilitating the recruitment of VCP.

Furthermore, we investigated the recruitment of VCP to SGs in HEK293T cells with or without OTUD6B knockdown. As shown in Fig. 8A, VCP demonstrated diffused staining with punctate structures dispersed in the cytoplasm in cells with or without OTUD6B knockdown under untreated conditions, where G3BP1 was evenly distributed in the cytosol. However, in cells treated with arsenite, VCP was evidently localized to G3BP1-positive SGs in control cells, while the SG recruitment of VCP was dramatically diminished in cells with OTUD6B knockdown (Fig. 8B). Given that results from our rescue experiments suggest impaired recovery of SG formation by catalytically inactive OTUD6B, we compared the VCP association of wild-type and inactive OTUD6B by performing co-IP assays, with results showing that the catalytic mutation C158A rendered OTUD6B attenuated VCP interaction (Fig. 8C, D and Supplementary Fig. 5). Therefore, our results collectively uncover the regulatory roles of OTUD6B in SG dynamics through facilitating SG coalescence of VCP.

## Discussion

SG formation is a conserved mechanism adopted by eukaryotic cells in response to environmental stresses, enabled by the assembly of a wide range of protein and RNA molecules through liquid-liquid phase separation [37–40]. In this study, we reveal that OTUD6B is recruited to SGs following arsenite and heat shock treatment. Functionally, we observed that OTUD6B promoted the early assembly of SGs as well as facilitated disaggregation after stress relief in a manner partially dependent on its enzymatic activity. Mechanistically, our combined analysis from interactomics and proximity proteomics demonstrates that OTUD6B regulates SG dynamics through association with VCP.

VCP/p97 is an evolutionarily conserved hexameric ATPase, best known for its role in maintaining cellular homeostasis by orchestrating protein turnover pathways such as ER-associated degradation (ERAD), autophagy, and endosomal trafficking [41–44]. Each subunit of VCP contains four characteristic domains: a regulatory N-terminal domain, two ATP-binding domains, in addition to linker regions and a disordered C-terminal tail [45]. Due to the functional diversity, specific turnover of substrates by VCP/p97 heavily relies on a large number of cofactors. To date, more than 40 cofactors have been reported to interact with the N- or C-terminus of VCP [46]. The emerging roles of VCP in regulating SG dynamics are not fully elucidated but have attracted growing attention. Although a previous study proposed that VCP might directly act on SGs to promote their clearance, FAF2 was recently reported to be a cofactor recruiting VCP to heat stress-induced SGs to extract ubiquitylated G3BP1, leading to granule disassembly [11, 46–49]. In addition to promoting SG recovery from heat shock, VCP was also shown to be recruited by ZFAND1 in arsenite-induced SGs to promote disassembly [50]. Therefore, the ability of VCP to promote SG clearance likely involves various cofactors. In the present study, we reveal OTUD6B as a novel VCP-associated factor that assists VCP in orchestrating SG dynamics. Intriguingly, ASPL, a well-known VCP cofactor, was also recently found to regulate the assembly and disassembly of SGs [51, 52]. Of note, our observations show that the depletion of VCP and ATPase inhibition by CB-5083 led to similar defects in SG dynamics, affecting both the early assembly as well as the disassembly of SGs induced by arsenite and heat shock. These findings suggest the importance of VCP ATPase activity in the overall SG dynamics.

Our findings reveal that OTUD6B functions to recruit VCP to SGs, promoting not only their early assembly, but also disassembly following stressor removal (Fig. 8E). Importantly, this regulation was observed with both arsenite and heat shock treatment, thus differing clearly from other VCP cofactors that respond to specific stimulus. This feature of OTUD6B is likely conferred by its association with the 40S ribosomes that are pivotal SG components. Besides OTUD6B, several DUBs were shown to regulate SG dynamics. USP10 plays a dual role in ribosome rescue and granule assembly [53]. In addition to protecting 40S subunits from degradation via deubiquitylation, USP10 also competitively binds to G3BP1 to suppress SG formation independent of enzymatic activity [54, 55]. Interestingly, another OTU family member, OTUD4, was previously identified as an SG protein and proposed to function in SG assembly [56]. It is noteworthy that both OTUD6B and OTUD4 have been reported to participate in antiviral responses in humans [17]. The specific roles of the two OTU proteins in SG dynamics warrant further investigation to elaborate.

In summary, this study demonstrates that OTUD6B functions as an important regulator of SG dynamics and homeostasis. Through the VCP association, OTUD6B accelerates the formation and disaggregation of SGs in response to different stressors. Biallelic variants in *OTUD6B* show intellectual disability syndrome associated with seizures, dysmorphic features, and impaired proteasome function [18]. The evidence that OTUD6B regulates SG dynamics, coupled with reported roles in proteostasis, calls for further investigation on this DUB, considering the vital roles of toxicity induced by protein aggregates and impaired SG dynamics in neurodegeneration [57, 58]. Therefore, our findings will shed light on potential therapeutic approaches through enhancing VCP-mediated SG disassembly. Given that defects in OTUD6B have been linked to various diseases, further research into its long-term physiological and pathological roles in animal models is also required to deepen our understanding of the complex SG biology, in addition to providing a basis to develop novel therapeutic strategies.

## Materials and methods

### Cell culture and stable cell lines

HeLa and HEK293T cells were purchased from ATCC and cultured in Dulbecco’s Modified Eagle’s Medium (DMEM, MeilunBio) supplemented with 10% fetal bovine serum (FBS, BI). Cells were maintained at 37 °C in a humidified incubator (Thermo, 3111) with 5% CO_2_. Stable cell lines were generated by lentiviral infection as described previously [59]. Briefly, HEK293T cells were transfected with vector and packing plasmids psPAX2 and pMD2G using jetPRIME® reagents. After 48 h, lentiviruses were collected and syringe-filtered before being added to cells. Positive clones were selected using 2 μg/mL of puromycin. Cells were routinely tested for mycoplasma contamination.

### Immunofluorescence

Cells were fixed with 4% paraformaldehyde (Beyotime) in PBS for 15 min, permeabilized with 0.2% Triton X-100 for 5 min, and blocked with 5% BSA for 30 min. Samples were incubated with the following primary antibodies: G3BP1 polyclonal antibody (Proteintech, 13057–2-AP), CoraLite® Plus 488-conjugated G3BP1 monoclonal antibody (Proteintech, CL488-66486), VCP monoclonal antibody (Proteintech, 60316-1-Ig), VCP recombinant antibody (Proteintech, 82463-1-RR), OTUD6B polyclonal antibody (Proteintech, 25430-1-AP), DYKDDDDK tag monoclonal antibody (anti-FLAG® tag) (Proteintech, 66008-4-Ig). After incubation with primary antibodies, cells were washed three times with PBS and incubated with secondary antibodies (594-conjugated goat anti-rabbit IgG (ABclonal, AS039) or Alexa Fluor® 488-labeled secondary antibodies (Abcam, ab150113)) and DAPI (Beyotime, C1002) for 40 min at room temperature. The coverslips were then washed with PBS and mounted. Imaging was performed using Olympus FV3000, and results were analyzed using Olympus FV31S-SW Viewer and FV31S-DT (Ver. 2.6) software.

### Immunoprecipitation and mass spec

Cells were lysed using the NP40 buffer as described previously [60]. Same amounts of lysate from each condition were incubated with prewashed protein A/G magnetic beads (MedChemExpress) along with specific antibodies for overnight at 4 °C. After incubation, beads were washed, and bound proteins were eluted in SDS-PAGE sample buffer for subsequent analyses. For immunoprecipitation of endogenous OTUD6B, cells were lysed in the presence of 100 μg/mL of RNase. Protein samples were subjected to reduction (10 mM DTT at 56 °C for 1 h) and alkylation (55 mM iodoacetamide at room temperature for 25 min). Samples were digested overnight with trypsin (Sequencing Grade, Promega) at 37 °C. After digestion, samples were acidified to pH < 3 using formic acid to a final concentration of 1%. Peptides were desalted using C18 Stage Tips, dried, and stored for mass spec analysis.

### APEX2 proximity labeling

HEK293T cells stably expressing APEX2-tagged OTUD6B fusion protein were seeded in T150 flasks. APEX2 proximity labeling was conducted by incubating cells in medium supplemented with Biotin-phenol (BP, Aladdin, 41994-02-9) at 500 μM for 30 min followed by addition of H_2_O_2_ (Sigma-Aldrich, H1009-100ML) at 1 mM for 1 minute, while negative control samples were not treated with H_2_O_2_. Cells were washed with fresh quencher solution (10 mM sodium azide, 10 mM sodium ascorbate, 5 mM Trolox) and lysed in ice-cold RIPA buffer supplemented with PMSF, quencher, and protease inhibitor for 15 min. Streptavidin magnetic beads (MedChemExpress, HY-K0208) were added into cleared lysates at a ratio of 20 μL of beads per 1 mg of proteins, before incubation with rotation for overnight at 4 °C. Beads were washed to remove nonspecific binders. Samples were suspended in 8 M urea/50 mM Tris buffer and processed for DTT reduction and IAA alkylation. Proteins were trypsin-digested and acidified. Peptides were desalted, dried, and stored at –80 °C.

### Antibody-guided proximity labeling (Ab-PL)

Stable OTUD6B knockdown and control cells were treated with 500 μM of NaAsO₂ for 15 min and fixed in 4% paraformaldehyde. Cells were washed with PBST and quenched in 1.25 M glycine for 5 min, before permeabilized with 0.5% Triton X-100. Endogenous peroxidases were neutralized by adding H_2_O_2_ to a final concentration of 1.5% for 1 h. After 5% goat serum treatment, samples were incubated with G3BP1 antibody (1:100, 13057-2-AP) in 3% goat serum at 4 °C overnight. After washing, cells were incubated with HRP-conjugated goat anti-rabbit antibody (1:500, K1223, ApexBio) at 4 °C overnight. After washing, cells were incubated in the reaction buffer (35% sucrose, 1.6 μM BP, 1.5 mM DTT, and 1 mM H_2_O_2_ in PBS) for 10 min at room temperature. The reaction was stopped in the quenching solution, and samples were washed three times before being harvested by scraping. The labeled samples were lysed in 4% SDS, sonicated using a Bioruptor, and de-crosslinked at 100 °C for 1 h. The lysate was centrifuged at 1000 × *g,* and the supernatant was mixed with ice-cold acetone for protein precipitation at –30 °C overnight. Protein was harvested by centrifugation at 10,000 × *g* for 3 min, and resuspended in RIPA buffer. 500 μg of proteins per condition were used for enrichment with streptavidin beads by incubation for 2 h at room temperature. Beads were resuspended in 2 M of urea to wash 4 times, followed by washes with PBS and 100 mM of ammonium bicarbonate. Proteins were reduced and alkylated before being digested by trypsin as aforementioned. Peptides were acidified with trifluoroacetic acid, desalted, and freeze-dried for storage.

### LC-MS and data analysis

Peptides were resuspended in 0.1% FA and loaded onto a column packed in-house with C18 particles (ReproSil-Pur 120 C18-AQ, Dr. Maisch). Samples were analyzed using a Q-ExactivePlus (ThermoFisher) coupled to an Easy nLC1000 system (ThermoFisher). Peptides were separated with the following gradient: 12–35% B for 60 min, 35–45% B for 10.5 min, 45–90% B for 0.5 min, 90% B for 9 min, 2% B for 10 min (A: 0.1% formic acid, B: 0.1% formic acid in 80% acetonitrile). The Q-Exactive Plus was operated in data-dependent mode with a full MS scan at a resolution of 60,000 followed by high-energy collision dissociation (HCD) with 27% normalized collision energy. MS2 spectra were acquired in the ion trap with an AGC target value of 15,000. Raw files were searched against the non-redundant UniProt human database using Open-pFind. Data processing and statistical analysis were performed using Perseus (v1.5.8.5). For APEX2-MS and Ab-PL, peptides were resuspended in 0.1% FA and analyzed using a Q-Exactive HF mass spectrometer (ThermoFisher) coupled to a Dionex UltiMate 3000 RSLCnano system (ThermoFisher) with a C18 reverse-phase column (150 μm inner diameter, 3 cm length, 1.9 μm resin). Peptide separation was performed with the following gradient: 4–12% B for 2 min, 12–32% B for 69 min, 32–45% B for 10 min, 45–90% B for 0.5 min, and 90% B for 9 min. The Q-Exactive HF was operated in data-dependent mode with a full MS scan (350–1750 m/z) at a resolution of 60,000 followed by HCD with 27% normalized collision energy. MS2 spectra were acquired in the ion trap with an AGC target value of 15,000. For APEX2-MS, raw files were searched against the non-redundant UniProt human database using MaxQuant, while raw files were searched using Open-pFind for Ab-PL.

### Statistics

Fluorescence intensity was quantified by tracing randomly selected regions of interest, and Pearson correlation was performed for signals between different channels. SGs were automatically counted using ImageJ from random microscopic views. Three biological replicates were designed to allow statistical analysis in GraphPad Prism version 8 software. Comparisons between two means were performed using a two-tailed Student’s *t* test. The statistical significance is denoted by an asterisk, where *P < 0.05 was considered significant.

## Supplementary information

Supplementary Figure legends

Supplementary Figure 1

Supplementary Figure 2

Supplementary Figure 3

Supplementary Figure 4

Supplementary Figure 5

## Data Availability

The data used and/or analyzed during the current study are included in this published article.
