# Correction: Proximity proteomics reveals OTUD6B regulation of stress granule dynamics through coalescence with VCP/p97

**DOI:** 10.1038/s41419-026-08842-7

**Published:** 2026-05-19

**Authors:** Dian Yang, Yichao Liu, Yueshun Hong, Enming Miao, Peng Wang, Yuming Sun, Lina Zhou, Shuyan Liu, Yingqiu Zhang, Hongqiang Qin, Mingliang Ye, Han Liu

**Affiliations:** 1 The Institute of Cancer Stem Cell, Dalian Medical University, Dalian, China; 2 State Key Laboratory of Medical Proteomics, Dalian Institute of Chemical Physics, Chinese Academy of Sciences, Dalian, China; 3 State Key Laboratory of Fine Chemicals, School of Chemical Engineering, Dalian University of Technology, Dalian, Liaoning China; 4 Instrumental Analysis Center, Dalian University of Technology, Dalian, China; 5 University of Chinese Academy of Sciences, Beijing, China

**Keywords:** Organelles, Proteomics

Correction to: *Cell Death & Disease* 10.1038/s41419-026-08451-4, published online 06 February 2026

In this article Fig. 2 and 7 have not been correctly displayed online.

**Figure 2** 
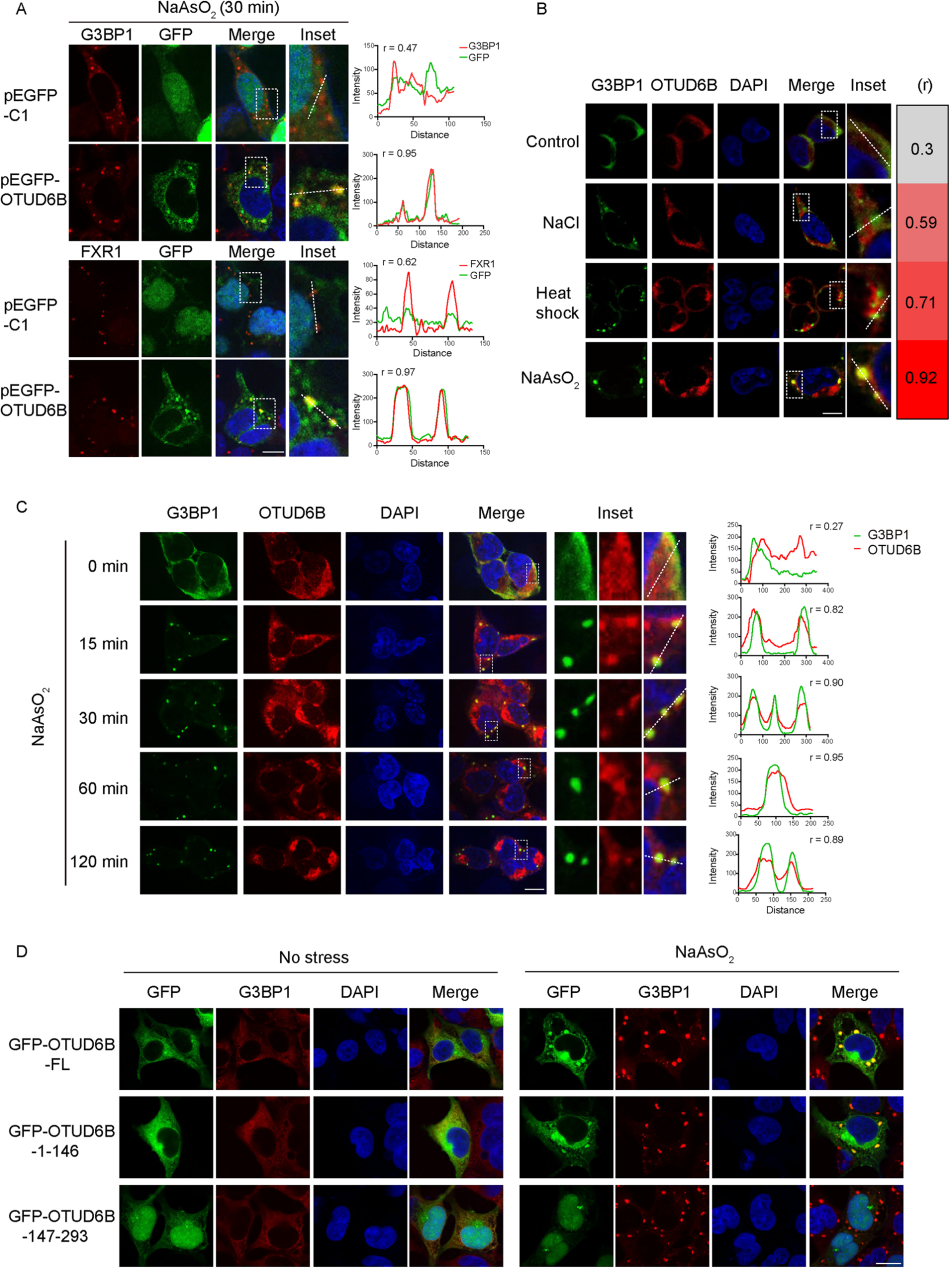


**Figure 7** 
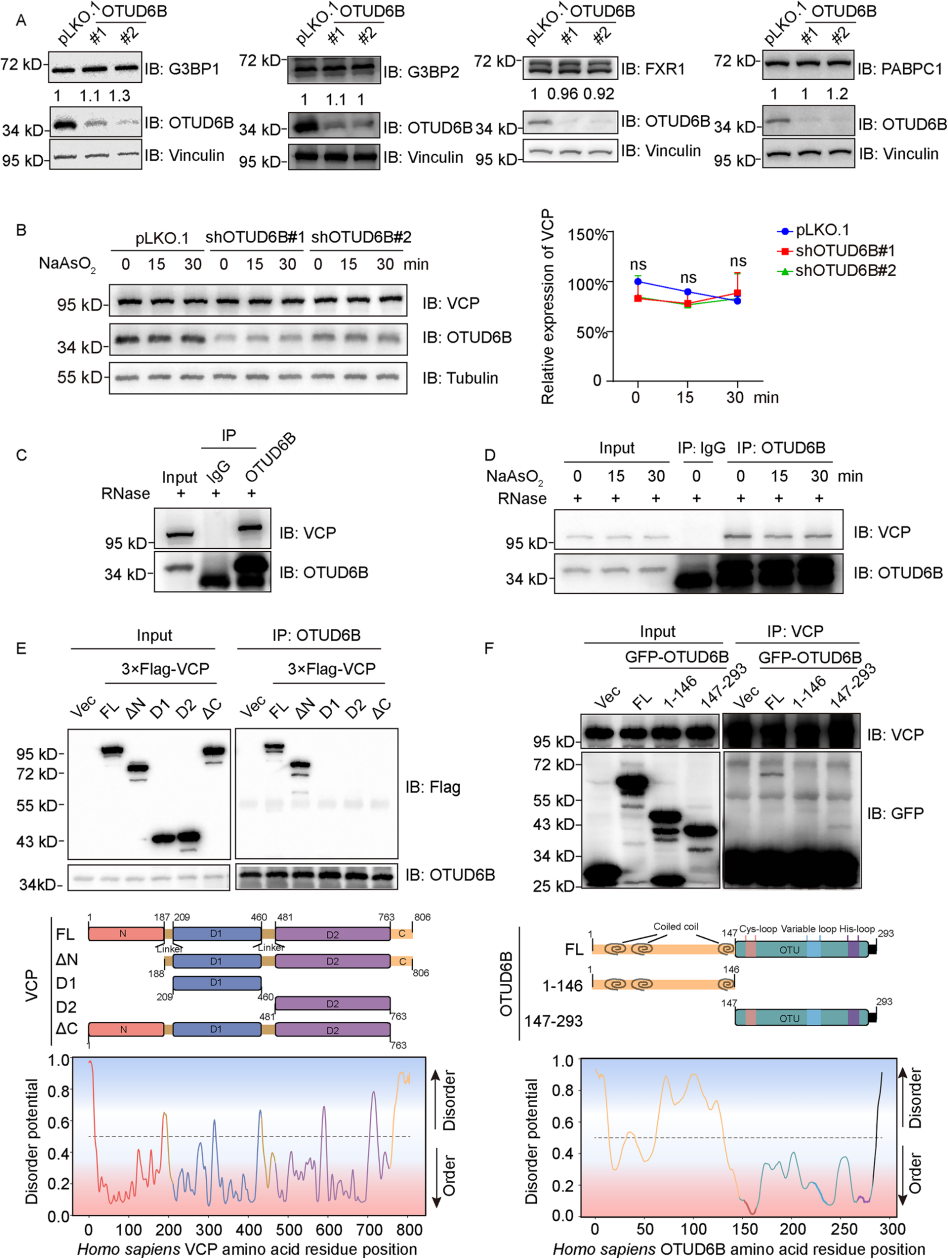


The original article has been corrected.

