# Supplementary material for: Proximity proteomics reveals OTUD6B regulation of stress granule dynamics through coalescence with VCP/p97
Source: Cell Death Dis. 2026 Feb 6;17(1):206. doi: 10.1038/s41419-026-08451-4 (PMC12894854; doi:10.1038/s41419-026-08451-4)
Supplement: Supplementary file 1 — Supplementary Figure legends [file 41419_2026_8451_MOESM1_ESM.docx]

**Proximity proteomics reveals OTUD6B regulation of stress granule dynamics through coalescence with VCP/p97**

Dian Yang^1,2^, Yichao Liu^3^, Yueshun Hong^1^, Enming Miao^2,4^, Peng Wang^1^, Yuming Sun^5^, Lina Zhou^5^, Shuyan Liu^1^, Yingqiu Zhang^1^, Hongqiang Qin^2,6^ *, Mingliang Ye^2, 4^*, Han Liu^1^*

**Supplementary figure legends**

**Supplementary Figure 1. OTUD6B associates with stress granule.**

A-C, HEK293T cells were lysed for immunoprecipitation (IP) assays using α-OTUD6B and IgG isotype control antibodies. IP and lysate input samples were subjected to immunoblotting analyses using antibodies against OTUD6B and indicated core SG proteins (G3BP1, G3BP2, FXR1, and CAPRIN1). D, Representative confocal images show the localization of endogenous OTUD6B and G3BP1 in HeLa cells treated by arsenite (500 μM) for indicated times. DAPI was used for nucleus staining. Corresponding co-localization analysis of the indicated fluorescence signals with Pearson’s correlation coefficients (r) is presented in the graph on the right. Inset shows magnified view of dashed rectangle-circled ROI (region of interest) in each group. Scale bar, 10 μm.

**Supplementary Figure 2. OTUD6B silencing decelerates arsenite-induced SG formation in HeLa cells.**

A, Representative immunoblots show shRNA-mediated knockdown of OTUD6B in HeLa cells stably transfected with two shOTUD6B (#1 and #2) and control pLKO.1 constructs. Vinculin was probed as loading control. Column chart shows the quantitation of relative expression levels of OTUD6B from independent experiments (n = 5). B, Representative confocal images show arsenite-induced SG formation by G3BP1 staining in HeLa cells with or without OTUD6B silencing. The stable cell lines were exposed to the indicated times of arsenite. Quantitation of cells with SGs for each group from independent experiments (n = 3) is presented on the right. Scale bar, 10 μm. Five random fields of view from each group were used for statistical analysis. Data are shown as mean ± SD. Student’s t-test was used to calculate *P* values. **P* < 0.05 and ns for statistically not significant.

**Supplementary Figure 3. OTUD6B promotes the early assembly of SG upon heat shock.**

A, Representative confocal images show the influence of GFP-tagged OTUD6B on heat shock-stimulated SG formation in HEK293T cells. HEK293T cells transfected with pEGFP-OTUD6B or empty vector (pEGFP-C1) as control were exposed to the indicated times of heat shock. IF staining of G3BP1 (red) and GFP signals (green), along with DAPI for nucleus (blue) were observed. B, Quantitation of cells with SGs in GFP-positive cells for each group in A at indicated time periods from independent experiments (n = 3). C and D, Representative confocal images show the effects of stable OTUD6B knockdown on heat shock-induced SG formation in HEK293T (C) and HeLa (D) cells. The stable cells were exposed to the indicated times of heat shock (43°C), and fixed for IF staining of G3BP1. Quantitation of cells with SGs for each group from independent experiments (n = 3) is presented on the right. E, Representative confocal images from rescue experiments in stable HEK293T cells. OTUD6B knockdown cells were transfected with either wild-type (WT) or catalytically-dead (C158A) form of GFP-tagged OTUD6B as indicated. SGs were induced by heat shock (43°C for 15 minutes). IF staining of G3BP1 (red) and GFP signals (green), along with DAPI for nucleus staining (blue) were observed. Quantitation of cells with SGs for each group from independent experiments (n = 3) is shown on the right. Scale bar, 10 μm. Five random fields of view from each group were used for statistical analysis. Data are shown as mean ± SD. Student’s t-test was used to calculate *P* values. ***P* < 0.01, ****P* < 0.001, and ns for statistically not significant.

**Supplementary Figure 4. VCP inhibition impairs the early formation of heat shock-induced SG.**

A, Representative confocal images demonstrate SG formation by G3BP1 staining in HEK293T cells with or without VCP silencing with two siRNAs as indicated. Cells were exposed to the indicated times of heat shock (43°C). Quantitation of cells with SGs for each group from independent experiments (n = 3) is shown on the right. B, Representative confocal images show SG formation in HeLa cells with pharmacological inhibition of VCP. Cells were pretreated with DMSO or CB-5083 (5 μM) for 4 h, before exposed to 15 or 30 min of heat shock (43°C). Quantitation of cells with SGs at the indicated time periods for each group from independent experiments (n = 3) is presented on the right. C, Representative confocal images show SG formation in HEK293T cells with or without stable OTUD6B knockdown in the presence of CB-5083. Cells were pretreated with CB-5083 (5 μM, 4 h), and then subjected to heat shock (43°C) for the indicated time periods. Cells were fixed for IF staining of G3BP1. Quantitation of cells with SGs at the indicated time periods for each group from independent experiments (n = 3) is shown on the right. Scale bar, 10 μm. Five random fields of view from each group were used for statistical analysis. Data are shown as mean ± SD. Student’s t-test was used to calculate *P* values. **P* < 0.5, ****P* < 0.001, ns for statistically not significant.

**Supplementary Figure 5. Uncropped original immunoblots for images in corresponding figures.**
