# Supplementary figures and images for: Proximity proteomics reveals OTUD6B regulation of stress granule dynamics through coalescence with VCP/p97
Source: Cell Death Dis. 2026 Feb 6;17(1):206. doi: 10.1038/s41419-026-08451-4 (PMC12894854; doi:10.1038/s41419-026-08451-4)

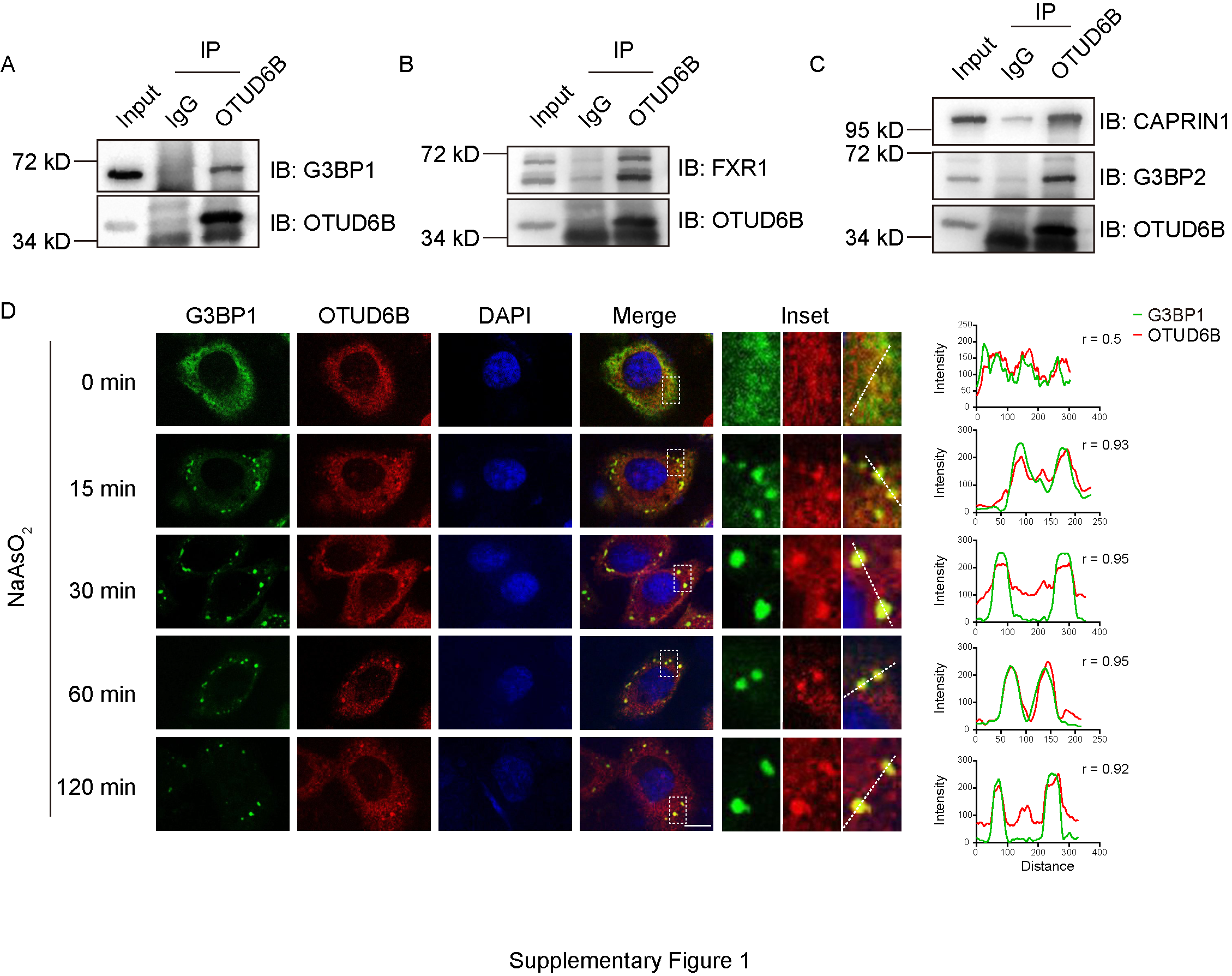

Supplement: Supplementary file 2 — Supplementary Figure 1 [file 41419_2026_8451_MOESM2_ESM.tif]

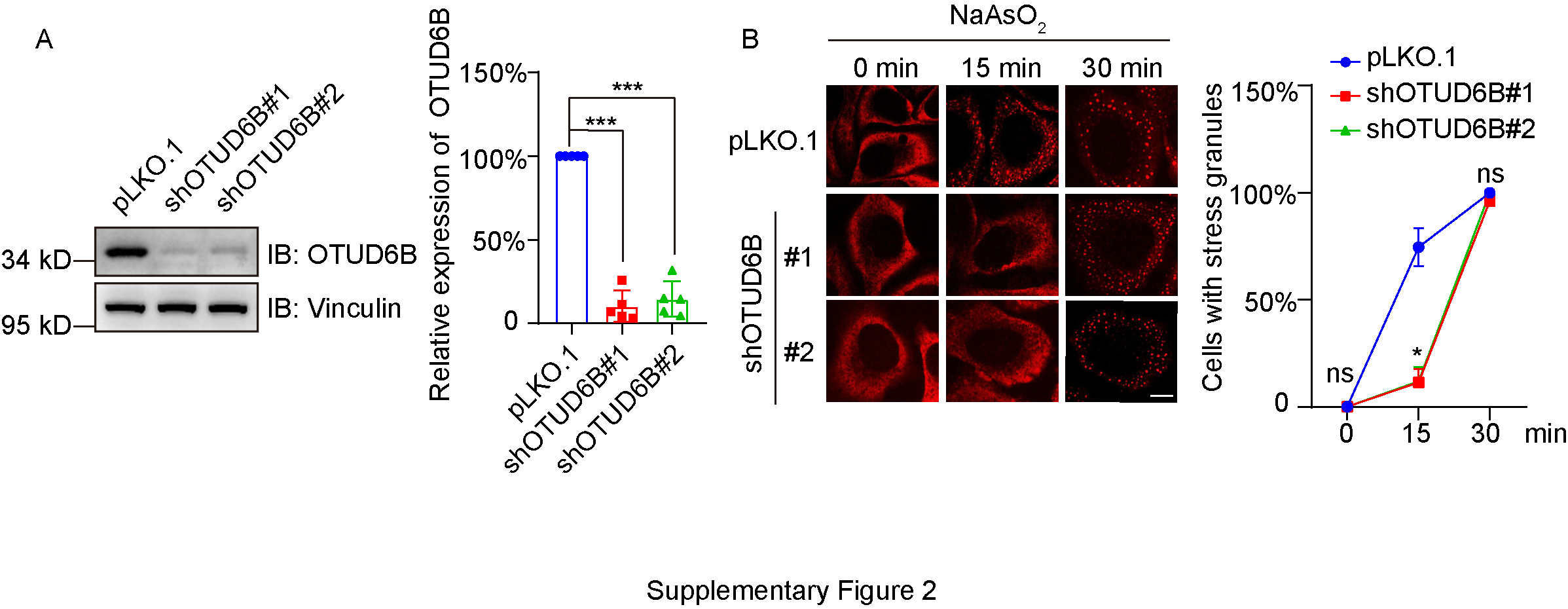

Supplement: Supplementary file 3 — Supplementary Figure 2 [file 41419_2026_8451_MOESM3_ESM.tif]

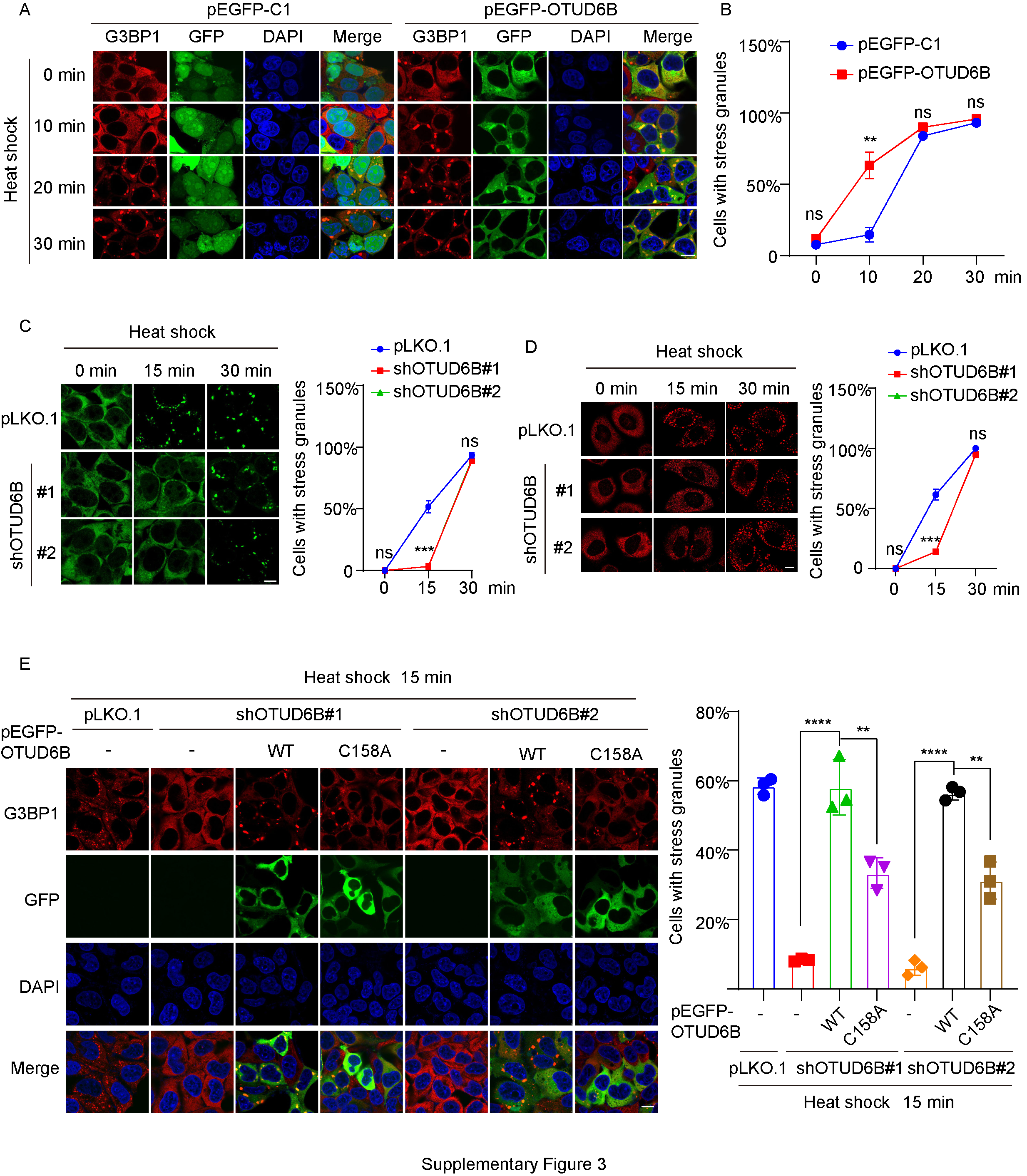

Supplement: Supplementary file 4 — Supplementary Figure 3 [file 41419_2026_8451_MOESM4_ESM.tif]

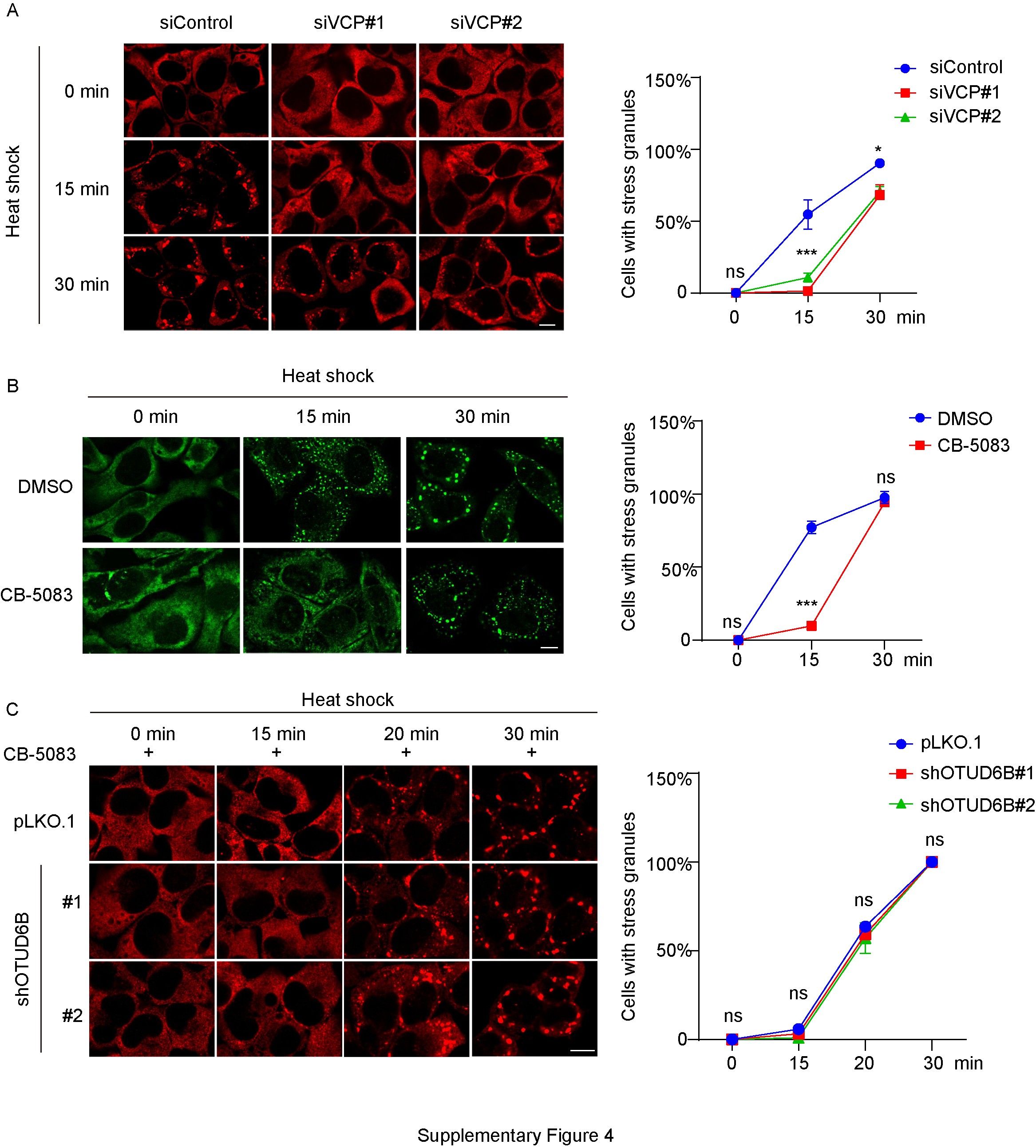

Supplement: Supplementary file 5 — Supplementary Figure 4 [file 41419_2026_8451_MOESM5_ESM.tif]
