# Supplementary material for: Proximity proteomics reveals OTUD6B regulation of stress granule dynamics through coalescence with VCP/p97
Source: Cell Death Dis. 2026 Feb 6;17(1):206. doi: 10.1038/s41419-026-08451-4 (PMC12894854; doi:10.1038/s41419-026-08451-4)

Figure3B

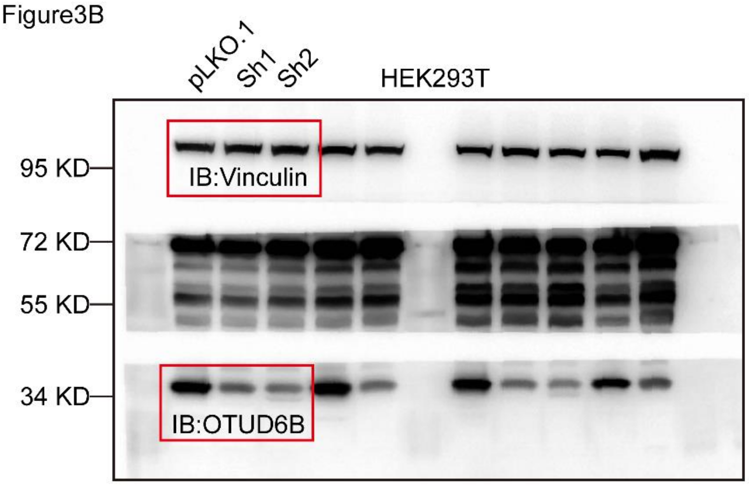

Figure6A

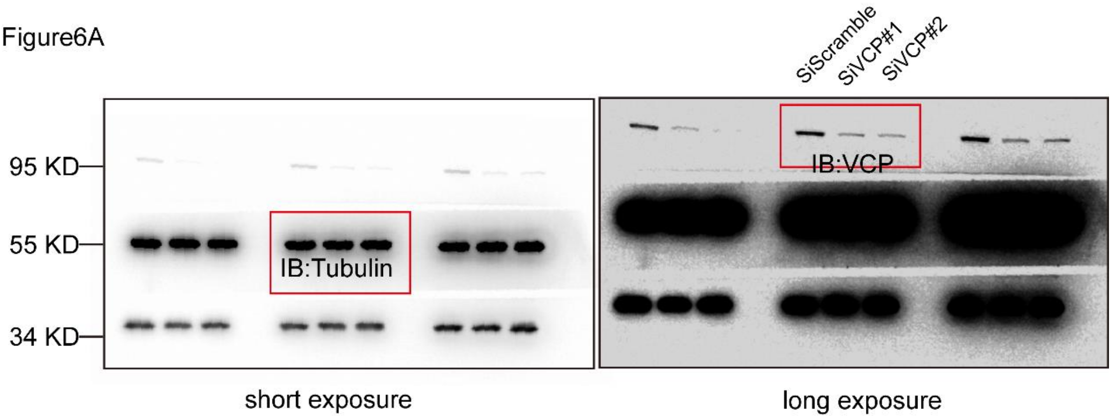

Figure7A

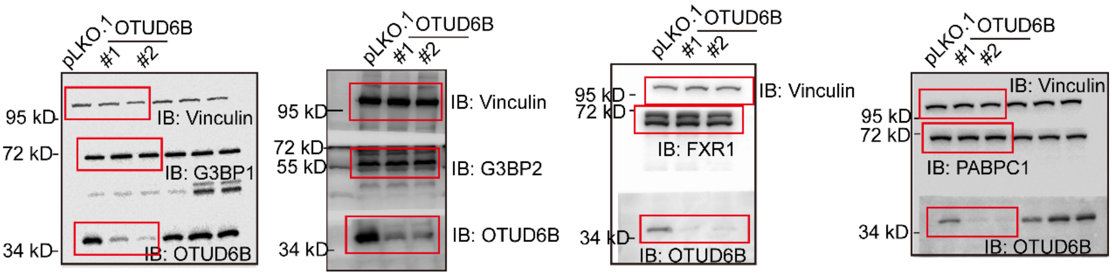

Figure7B

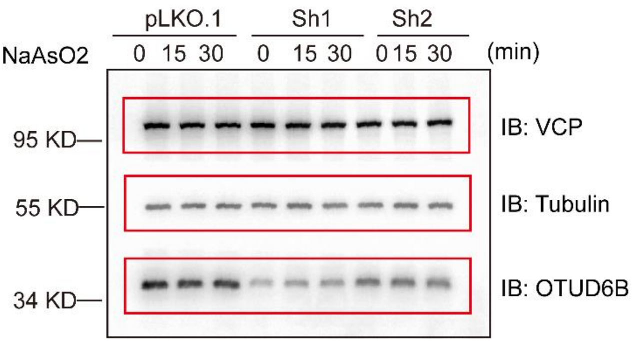

Figure7C

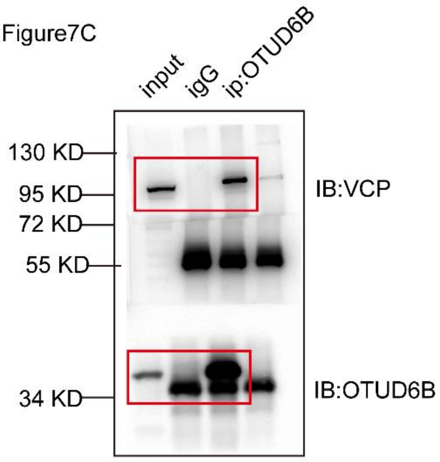

Figure7D

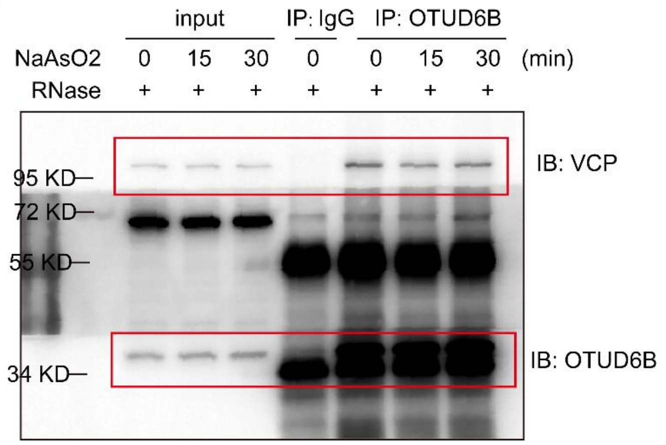

Figure7E

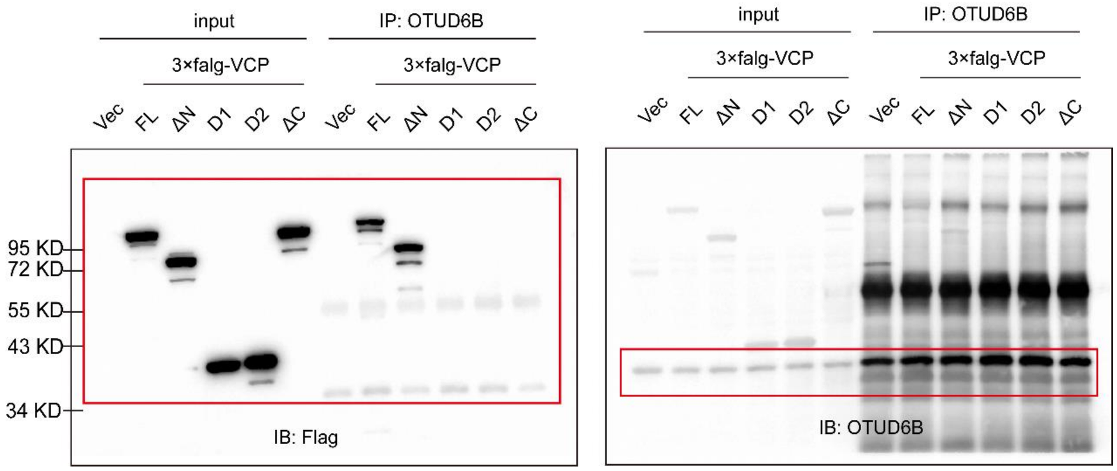

Figure7F

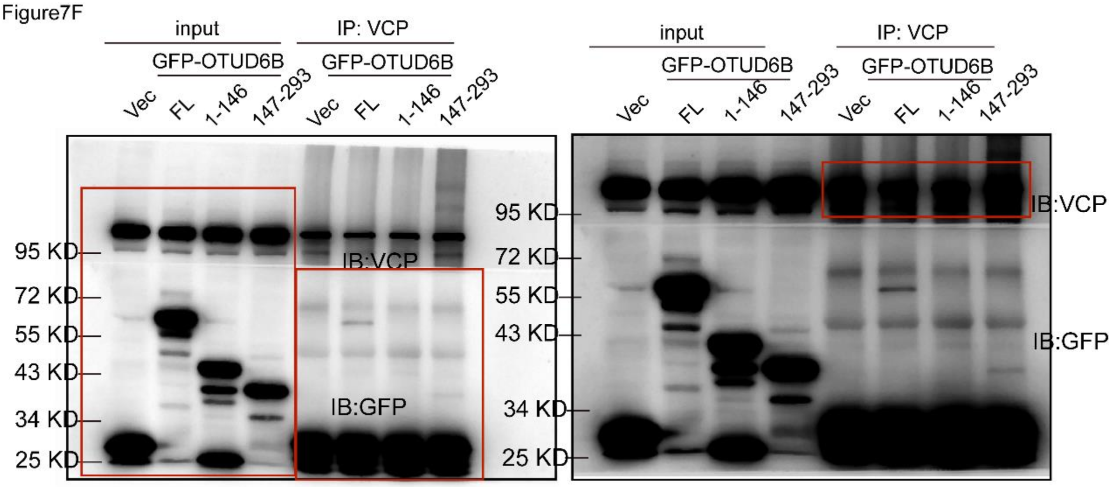

short exposure

Long exposure

Figure8C

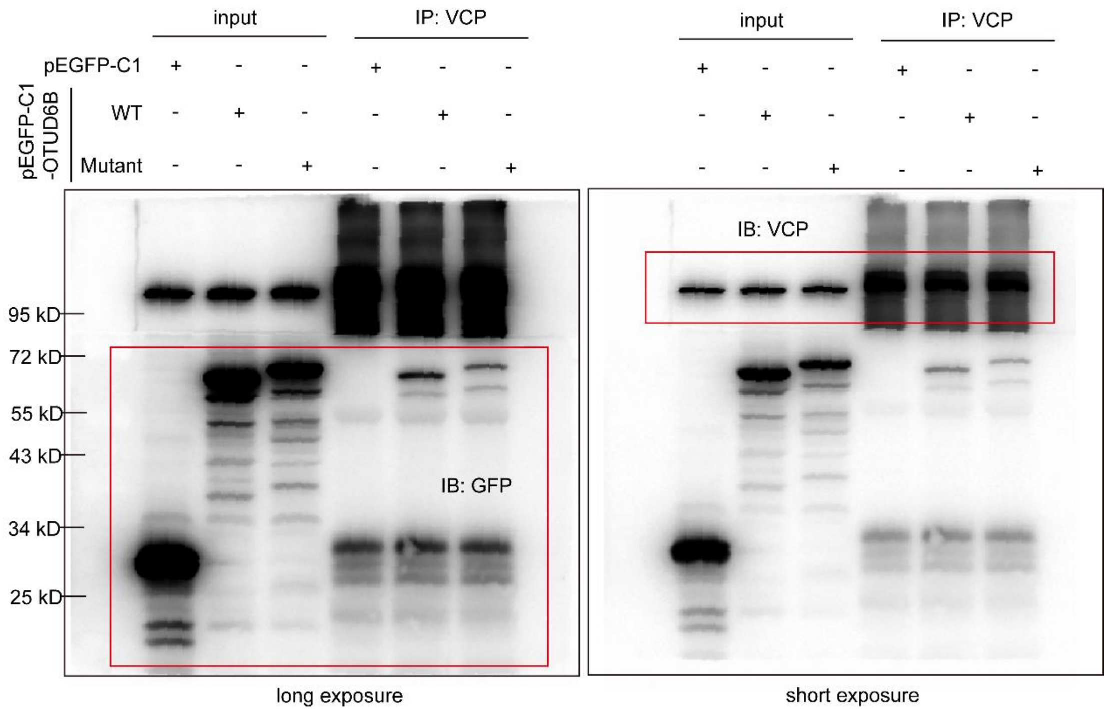

Supplementary Figure1

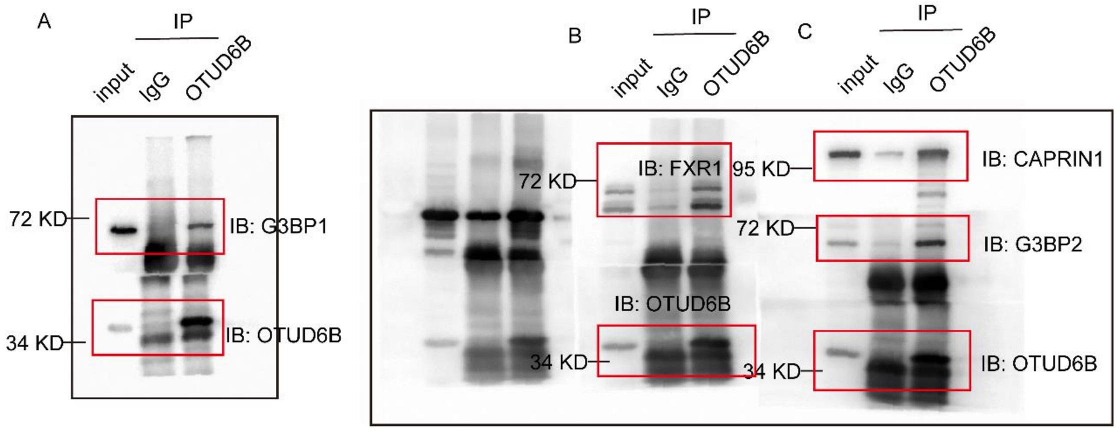

Supplementary Figure2A

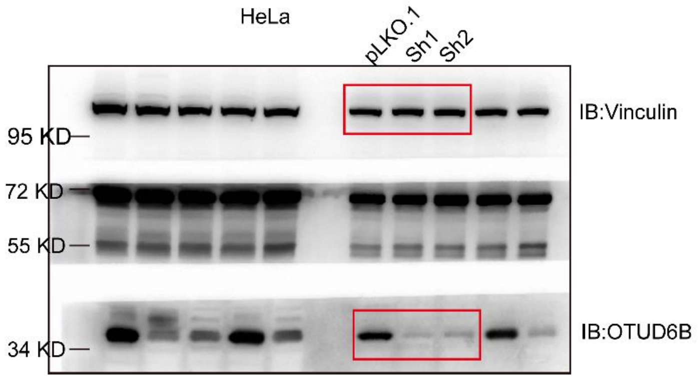

Supplement: Supplementary file 6 — Supplementary Figure 5 [file 41419_2026_8451_MOESM6_ESM.pdf]
